# Carnosine's Effect on Amyloid Fibril Formation and Induced Cytotoxicity of Lysozyme

**DOI:** 10.1371/journal.pone.0081982

**Published:** 2013-12-11

**Authors:** Josephine W. Wu, Kuan-Nan Liu, Su-Chun How, Wei-An Chen, Chia-Min Lai, Hwai-Shen Liu, Chaur-Jong Hu, Steven S. -S. Wang

**Affiliations:** 1 Department of Optometry, Central Taiwan University of Science and Technology, Taichung, Taiwan,; 2 Department of Chemical Engineering, National Taiwan University, Taipei, Taiwan; 3 Department of Neurology, Shuang Ho Hospital, Taipei Medical University, New Taipei City, Taiwan; Aligarh Muslim University, India

## Abstract

Carnosine, a common dipeptide in mammals, has previously been shown to dissemble alpha-crystallin amyloid fibrils. To date, the dipeptide's anti-fibrillogensis effect has not been thoroughly characterized in other proteins. For a more complete understanding of carnosine's mechanism of action in amyloid fibril inhibition, we have investigated the effect of the dipeptide on lysozyme fibril formation and induced cytotoxicity in human neuroblastoma SH-SY5Y cells. Our study demonstrates a positive correlation between the concentration and inhibitory effect of carnosine against lysozyme fibril formation. Molecular docking results show carnosine's mechanism of fibrillogenesis inhibition may be initiated by binding with the aggregation-prone region of the protein. The dipeptide attenuates the amyloid fibril-induced cytotoxicity of human neuronal cells by reducing both apoptotic and necrotic cell deaths. Our study provides solid support for carnosine's amyloid fibril inhibitory property and its effect against fibril-induced cytotoxicity in SH-SY5Y cells. The additional insights gained herein may pave way to the discovery of other small molecules that may exert similar effects against amyloid fibril formation and its associated neurodegenerative diseases.

## Introduction

Amyloid diseases, including hemodialysis amyloidosis, type II (or noninsulin-dependent) diabetes, Parkinson's disease, transmissible spongiform encephalopathies, Huntington's disease, and Alzheimer's disease, are a group of human diseases that are characterized by the formation of extracellular insoluble aggregates or deposits (also termed as amyloid fibrils) in certain tissues and organs [1]–[4]. While the precursor proteins involved in the aforesaid diseases share no sequence homology and native structural motif similarity, they form fibrillar aggregates with common morphological and histochemical features. For example, an ordered cross β-sheet-rich secondary structure, fibrillar morphology with a diameter of 5–15 nm, birefringence to polarized light upon interaction with Congo Red, fluorescence after reacting with thioflavin T (ThT), insolubility in most solvents, and resistance to protease degradation [1], [5], [6].

The formation of amyloid fibrils or fibril-like aggregates has also been observed in proteins that are not associated with any form of disease under certain environmental stresses (e.g., high temperature, extreme pH, vigorous agitation, and high pressure) [7]–[9]. Evidence has shown that the morphological, histochemical, and cytotoxic properties of the aggregates derived from these non-disease-related proteins are similar to those of disease-associated amyloid proteins, suggesting that amyloidogenicity or amyloid fibril-forming propensity is a generic property of all polypeptides [10], [11].

Hen egg-white lysozyme (HEWL), a 129-residue monomeric protein with molecular weight of 14.3 kD, has been extensively used as a food preservative owing to its lytic activity against the cell wall of Gram-positive bacteria [12], [13]. Structurally, HEWL, in its native conformation, is a helix-rich protein (α-helix: ∼30%) containing four disulfide bonds [14], [15]. Because of its well-defined structure information and a high degree of sequence and structural homology to the human lysozyme [16], [17], which is affiliated with familial lysozyme systemic amyloidosis [18], HEWL has been widely chosen as a model protein in research relating to the subjects of protein folding, unfolding, and aggregation. Numerous studies have demonstrated that HEWL is prone to fibrillate in a heated and acidic environment [17], [19], a concentrated ethanol solution [20], a concentrated solution of guanidine hydrochloride [21], and a vigorously agitated condition [22]. Therefore, hen egg-white lysozyme serves as a nice model system with which to study *in vitro* phenomena associated with fibril formation.

To combat amyloid diseases, efforts have been directed toward seeking or developing a variety of therapeutic strategies [23]–[25]. An increasing body of evidence points to the possible relation of fibrillar and/or protofibrillar species derived from amyloid proteins and the disease pathology; thus, considerable efforts are underway to screen small inhibitory molecules/compounds that are capable of counteracting the fibrillogenesis of disease-related amyloid proteins. A variety of natural or synthesized molecules and/or compounds have been reported to retard or prevent fibril formation both *in vitro* and *in vivo* [9], [26]–[28].

Carnosine, a naturally-occurring compound discovered by Gulevitch and Amiragdibi over 100 years ago [29], is found predominantly in human muscles, heart, liver, brain, kidneys, and other long-lived tissues. It has been regarded as one of the most common dipeptides in human and other mammals [30], [31]. Carnosine is synthesized from β-alanine and L-histidine, by the ATP-driven enzyme, carnosine synthetase, and is hydrolyzed by specific metal ion-dependent homodimeric dipeptidase (carnosinases) in the blood and other tissues [32]–[34]. In general, the intramuscular concentration of carnosine is found to be approximately 20–30 mM in human, about twenty-fold higher than that in mice, ten-fold higher than that in rabbits, and three-fold higher than that in cows, suggesting a high correlation between maximum life expectancy of various mammalian species and the concentration of carnosine present in the body [35]–[37].

Carnosine has been patented as an eye-drop component used for the treatment and prevention of senile cataract in Europe [38] and has also recently been discovered to have physiological and therapeutic functions (e.g., anti-aging and free-radical scavenging properties) in the human body [39]. Importantly, carnosine has been found to disaggregate glycated α-crystallin protein [40] and to prevent the native protein from forming amyloid fibrils [41]. In addition, carnosine has been shown to suppress the accumulation of β-amyloid in the central nervous system of transgenic mice model for Alzheimer's disease [42] and attenuate the *in vitro* fibrillogenesis of β-amyloid peptide Aβ(1–42) [43], [44]. *In vitro* cell culture studies demonstrated that carnosine can prevent rat brain endothelial cells [45] and rat PC12 cells from cytotoxic effects induced by β-amyloid peptides [46]. Based on another study, carnosine has also been shown to be effective in reducing the toxicity of prion protein possibly owing to its stimulatory effect in prion proteolysis and/or decreased glycolysis, which has been found to be upregulated by prion proteins and contributes toward protein glycoxidation [47].

Despite ample studies in the literature showing the effects of carnosine on human and animals, the exact mechanism of how this dipeptide exerts its effect (in particularly inhibitory activity against fibrillogenesis) remains elusive. In the present study, we present a more complete picture of of carnosine's mechanism of action in hen egg white lysozyme (HEWL) amyloid fibril inhibition from the molecular to cellular perspectives. We thoroughly examined the dose-dependent effect of carnosine on HEWL fibril formation from the protein structural level via a wide variety of methods, including several spectroscopic techniques (e.g., thioflavin T fluorescence spectroscopy, Congo red absorption spectroscopy, far-UV circular dichroism (CD) spectroscopy, and Nile red fluorescence spectroscopy) and transmission electron microscopy (TEM). The change in equilibrium thermal denaturation property of HEWL due to carnosine inhibition, as well as the important binding interactions by which the dipeptide exerts its inhibitory effect on amyloid fibrillogenensis, were also investigated. Finally, we explored the effect of carnosine in preventing SH-SY5Y cell death induced by HEWL amyloid fibril through a number of cell toxicity and viability tests (e.g., (4,5-dimethylthiazol-2-yl)2,5-diphenyltetrazolium bromide (MTT) reduction assay, lactate dehydrogenase (LDH) release assay, etc.).

## Materials and Methods

### Materials

Hen egg-white lysozyme (HEWL) (EC 3.2.1.17) was obtained from Merck (Germany). Hydrochloric acid and sodium chloride were purchased from Nacalai Tesque, Inc (Japan). Glycine was obtained from BioBasic, Inc (Canada). Carnosine and other chemicals, unless otherwise specified, were of analytical grade and purchased from Sigma (USA).

### Preparation of HEWL fibrils

All HEWL samples (∼10 mg/mL or ∼0.70 mM) without and with various concentrations of carnosine were prepared immediately prior to each experiment via dissolving HEWL powders in glycine buffer (100 mM glycine/HCl, pH 2.0, 100 mM sodium chloride and 1.54 mM NaN_3_), in accordance to previous studies [48], [49], prepared immediately prior to each experiment. In order to generate amyloid fibrils, each sample (10 mL) was transferred to a 20 mL glass tube and agitated with polytetrafluoroethylene (PTFE)-coated micro stirring bars (8 mm×1.5 mm) at ∼580 rpm in 55°C [50], [51]. At designated time points, the tube was gently vortexed to well-distribute the HEWL sample solution, and then an appropriate amount of HEWL sample was withdrawn for the following measurements.

### Thioflavin T (ThT) fluorescence measurements

The stock solution of ThT at a concentration of 1 mM was prepared in 95% (v/v) ethanol protected from light prior to use, and the concentration was determined spectrophotometrically using the molar extinction coefficient at 416 nm of 26600 M^−1^cm^−1^ [52]. Phosphate buffered saline (136.7 mM NaCl, 2.68 mM KCl, 0.01 M Na_2_HPO_4_, 1.76 mM KH_2_PO_4_, and 1.54 mM NaN_3_, pH 7.4) was used to dissolve the ThT stock solution to obtain a ThT working solution at a concentration of 10 µM. HEWL sample solutions (40 µL), with or without carnosine, taken out at different incubation times were mixed thoroughly with ThT working solution (960 µL) prior to measuring the ThT fluorescence emission intensities at 485 nm. The ThT fluorescence measurements were taken for 60 sec by exciting the resultant mixtures at 440 nm via Cary Eclipse fluorescence spectrophotometer (Varian, USA).

### Congo red (CR) binding assay

Fresh solution of CR dye with 10 µM in 100 mM phosphate buffer (pH 7.4) was diluted from the stock solution prior to use. The stock solution was prepared in phosphate buffer with 40% ethanol as described by Klunk *et al*. [53] and the concentration was determined spectrophotometrically using the molar extinction coefficient at 505 nm of 59300 M^−1^cm^−1^ [53]. Aliquots of 20 µL of fresh CR solution (10 µM) were added to 1980 µL of HEWL sample solutions followed by vortex-mixing for 15 s and incubating at room temperature for 30 min. The absorption spectrum of each sample was recorded over a range of 400–700 nm on a Cary 50 UV-Visible Spectrophotometer (Varian, USA) using a 1-cm path length quartz cuvette.

### Transmission electron microscopy (TEM) analysis

Aliquots of 5 µL of HEWL samples for TEM analysis were withdrawn from the working solutions used for ThT fluorescence measurements at 10 hr incubation and applied to a carbon-stabilized, formvar coated grid for 30 sec. Excess samples were removed by applying ashless filter papers to the edge of the grids and the grids were negatively stained with 2% uranyl acetate in distilled de-ionized water (Electron Microscopy Sciences, USA) for another 30 sec. After removing the excess stain, the grids were left to air-dry for at least 20 min and then examined and photographed on a Hitachi H-7650 transmission electron microscope with a Gantan model 782 CCD Camera (Tokyo, Japan) at an accelerating voltage of 100 kV.

### Far-UV circular dichroism (CD) spectroscopy

The secondary structural changes of HEWL sample solutions were evaluated by far-UV CD spectroscopy. CD spectra of HEWL samples were recorded after diluting 100-fold with de-ionized water over the wavelength range of 190–260 nm using a J-815 spectrometer (JASCO, Japan) with a 0.2 cm path length sample cell. All CD measurements were collected at room temperature using a bandwidth of 1.0 nm, a step interval of 0.1 nm, and a scanning speed of 50 nm/min. Each CD spectrum was the average of three scans. The secondary structure contents of HEWL samples were estimated using the CONTINLL algorithm with the reference set SP175 available from the DICROWEB website [54], [55].

### Nile red binding assay

Nile red was chosen as a hydrophobic fluorescent dye to avoid interference from Raman and Rayleigh scattering. Before measurement, a fresh working solution of Nile red was prepared by diluting the stock solution (1 mM dissolved in DMSO) to 1 µM with 100 mM glycine buffer. Aliquots of 20 µL of HEWL samples with and without carnosine taken at various time points were immediately mixed with 980 µL of working solution containing Nile red at 1 µM, which was found to be an optimum concentration for obtaining significant fluorescence changes without interference by the inner filter effect [56], [57]. The Nile red fluorescence emission spectra from 600 to 700 nm were measured at the excitation wavelength of 570 nm on a Cary Eclipse fluorescence spectrophotometer (Varian, USA) after at least 15 min incubation in the dark.

### Equilibrium thermal denaturation/unfolding and data analysis

Denaturation curves were constructed by measuring the intrinsic fluorescence of HEWL as a function of temperature, and then subsequently analyzed to yield the thermodynamic parameters for HEWL unfolding with and without carnosine. In thermal scans, the thermally-induced unfolding transition of HEWL samples was determined by monitoring the changes in intrinsic fluorescence intensity over 20–95°C with a heating rate of 1°C/min and an equilibrium time of 1 min. Intrinsic fluorescence emission spectra in the range of 300–420 nm were recorded every 2°C at the excitation wavelength of 280 nm on a Cary Eclipse fluorescence spectrophotometer (Varian, USA). The obtained thermally-induced unfolding data were analyzed by least-squares fitting against a two-state transition model via the procedure described as follows:

For a reversible protein unfolding reaction, the transition curve between the native and unfolded states is a function of temperature. The equilibrium constant (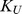) at any temperature could be calculated from equation (2).
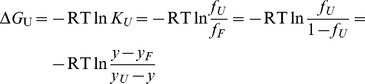
(1)where 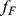 and 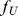 are the fractions of folded and unfolded protein species at various temperatures, respectively, y is the observed average emission wavelength (AEW) of HEWL intrinsic fluorescence spectrum. 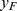 and 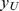 denote the pre- and post-unfolding baselines, which are assumed to be linearly dependent on heating temperature and thus can be represented by 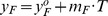 and 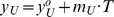, where 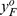, 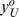, 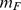 and 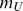 are the intercepts and slopes of the baselines before and after the transition, respectively, R is the gas constant, T is the temperature, and 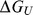 is the Gibbs free energy of thermal unfolding.

### Bioinformatic prediction of aggregation sites

Various aggregation site predictors using protein's primary sequence as input have been devised in recent years to predict “hot spots” or short sequences of amino acids that are prone to aggregation. Among these, AGGRESCAN [58], AMYLPRED [59], and TANGO [60] were utilized herein to calculate consensus potential aggregation sites on HEWL. AGGRESCAN predicts aggregation hot spots from an aggregation-propensity scale for amino acids derived from *in vivo* experiments that involved a highly amyloidogenic stretch in Aβ-42 peptide. AmlyPred incorporates a variety of algorithms to achieve a more objective prediction of aggregation sites. These algorithms include secondary structure [61], amyloidogenic pattern [62], β-aggregate tendency [60], packing density [63], and conformational energy [64] calculations. Of the three methods used, TANGO is the only one that accounts for solution parameters to simulate experimental conditions. We, therefore, set the prediction parameters to pH 2.0 and 55°C to match our experimental settings.

### Molecular docking of carnosine to lysozyme

Carnosine was first built using ChemDraw Ultra (Cambridge Soft Corp. Cambridge, MA) and optimized in three-dimensional form in Discovery Studios 2.5.5 (Accelrys Inc., San Diego, CA).

To be consistent with our experimental conditions, we subjected the X-ray structure (PDB code 2ZQ3) of HEWL to molecular dynamics (MD) simulations under pH 2.0 and 55°C. The system reached equilibration around 15 ns (see Fig. S1 of the supporting information) and a protein structure was extracted at 20 ns for the docking simulation.

Molecular docking using CDOCKER [65] was implemented to understand how carnosine inhibit HEWL fibril formation. CDOCKER is a molecular dynamics (MD) simulated-annealing-based algorithm incorporated into the Discovery Studios 2.5.5 environment. The grid-based molecular docking method employs CHARMm force field [66] to perform conformational searches for optimal binding modes of ligands to proteins or receptors followed by refinement of poses through minimization. Automated search for potential binding sites based on surface cavities and ligand volume yielded two potential binding sites on HEWL with the input site spheres defined with a grid extension of 8 Å and a radius of 12 Å from the center of ligand-binding site. The simulated annealing protocol consists of first heating the system to 427°C in 2000 steps, followed by annealing in 5000 steps to a final temperature of 55°C. For each potential binding site, one hundred random conformations were generated for carnosine based on the starting structure and allowed to be randomly distributed around the center of the sites. The final minimization step refined the docked poses and the ligand-protein interactions. The intramolecular energy for each ligand models were then computed to produce a total docking energy score (CDOCKER energy).

### Cell culture

Human neuroblastoma SH-SY5Y cells were maintained under humidified 5% (v/v) CO_2_/air at 37°C in a mixture of Dulbecco's modified Eagle's medium (DMEM) and Ham's F12 medium (F12) supplemented with 10% (v/v) FBS and 100 U/mL penicillin.

### 3,(4,5-Dimethylthiazol-2-yl)2,5-diphenyltetrazolium bromide (MTT) reduction assay

SH-SY5Y cells at a density of 1×10^5^ cells/mL were plated in the 96-well plates followed by 24 hr-incubation and various sample solutions were added to the cells for another 24 hr after plating. Cell viability of SH-SY5Y cells was measured using the MTT reduction assay. Viable cells reduce MTT to form blue formazan crystals, and inhibition of this reaction is indicative of the cellular redox changes that could result in cytotoxicity [67]. Stock sample solutions of HEWL with various concentrations of carnosine were diluted with sterile DMEM-F12 medium. The resultant diluted solutions were added to SH-SY5Y cells in the 96-well plates. Upon incubation for 6, 12, and 24 hr, the MTT reduction assay was performed after removal of the treatment. MTT was added to the culture medium to yield a final concentration of 0.5 mg/mL and incubated with cells for 4 hr in a CO_2_ incubator and 100 µL of a 5∶2∶3 N,N-dimethylforamide (DMF): sodium dodecylsulfate (SDS): water solution (pH 4.7) was added to dissolve the formazan crystals. After 18 hr of incubation in a humidified CO_2_ incubator, the absorbance at 585 nm for each well was measured using a µQuant Microplate reader (Bio-Tek Instruments, VT, USA). Cell viability was reported relative to control cells that were not exposed to the HEWL fibril solutions.

### Lactate dehydrogenase (LDH) release assay

The level of the cytoplasmic enzyme, lactate dehydrogenase, released from human neuroblastoma SH-SY5Y cells into DMEM/F12 culture medium upon cell membrane damage or lysis was determined for the quantification of cell death using an *in vitro* toxicology assay kit (Tox-7) (Sigma, USA) according to the instructions of the manufacturer. The reduction of NAD^+^ to NADH accompanied by the oxidation of lactate to pyruvate is catalyzed by LDH in the suspension. The resulting NAD^+^ is utilized in the stoichiometric conversion of a tetrazolium dye to a soluble colored formazan derivative, which strongly absorbs at 490–520 nm. Cells were seeded at a density of 1×10^5^ cells/mL in the 96-well plates for 24 hr-incubation under humidified 5% (v/v) CO_2_/air at 37°C and the aged HEWL sample without or with various concentration of carnosine was added to the cells for 6, 12, and 24 hr. The percentage of LDH release was defined as the ratio of LDH enzymatic activity of the group treated with aged HEWL sample to that of the group treated with lysis buffer (maximum LDH activity). LDH enzymatic activity measurements were performed following the procedures provided in the manufacturer's instructions (Sigma, USA).

### Flow cytometric analysis

Cells undergoing apoptosis were detected by double staining with Annexin V-FITC/PI in the dark according to the manufacturer's instructions (Sigma, USA). SH-SY5Y cells were seeded at a density of 1×10^6^ cells/mL in a 6-well plate. After 24 hr-incubation of cells in a humidified 5% CO_2_/air incubator and 37°C, the culture medium was replaced with 1 mL of medium containing HEWL with various concentrations of carnosine. Upon incubation in the same culture condition for 24 hr, cells were trypsinized and washed twice in cold phosphate buffered saline (PBS) under centrifugation (1400 rpm, 5 min). Cell pellets were suspended in Annexin V binding buffer at a concentration of 10^6^–10^7^ cells/mL. 100 µL of the resulting cell solution were first mixed with 10 µL of Annexin V-FITC for 15 min at 0°C in the dark, followed by the addition of 380 µL of Annexin V binding buffer and 10 µL of 50 µg/mL propidium iodide (PI). The stained cells were immediately analyzed using flow cytometry (Beckman Coulter FC500, USA). The fluorescence emission signals for annexin V-FITC were plotted against PI to identify viable cells (i.e. negative for Annexin V-FITC and PI signals), early apoptotic cells (Annexin V-FITC signal only) and late apoptotic/necrotic cells (positive for Annexin V-FITC and PI signals). Analysis was performed using Cytomics CXP Analysis software (Beckman Coulter, USA). Each measurement was carried out at least three times.

### Statistical analysis

All data presented are expressed as the mean ± the standard deviation (S.D.) of n independent determinations. Specific n values (n≥5) are reported in the figure legends. To determine whether a given data set was significantly different from a control data set, one tailed Student's t-test assuming unequal variances was performed. Unless otherwise noted, a criterion of p<0.05 was employed to determine whether the sample set and the untreated control set were statistically different. Asterisks above the bars indicate the significance of the result relative to the untreated control: a single asterisk indicates a value of p<0.05 and double asterisks indicate a value of p<0.01. The statistical analyses were conducted using Excel or KaleidaGraph software.

## Results

### HEWL fibrillogenesis was affected by carnosine as revealed by ThT fluorescence and Congo red binding

To assess the formation of fibrillar species at low pH (pH ∼2.0) and high temperature (55°C) under vigorous agitated condition (∼580 rpm), the tinctorial and ultra-structural properties of the HEWL samples were examined. The samples were first characterized by monitoring the changes in ThT fluorescence emission and Congo red absorption spectrum, then morphologically analyzed by TEM.

**Figure 1:**
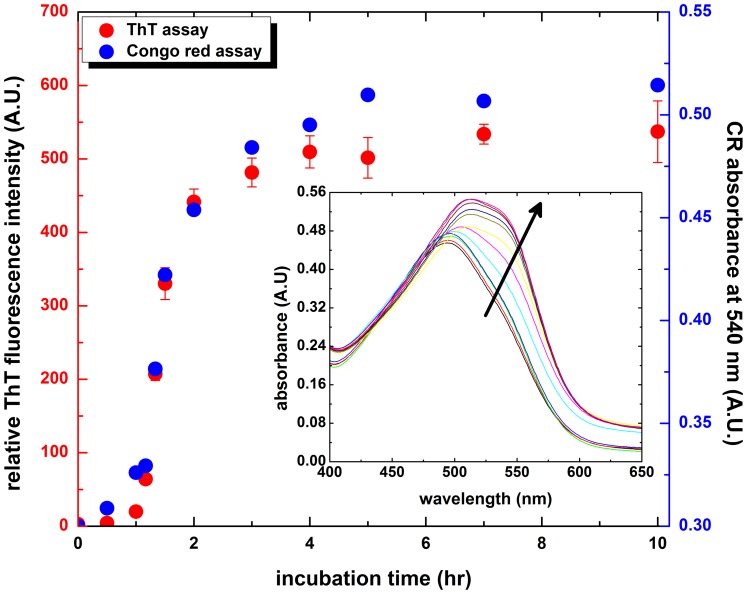
Amyloid fibrillogenesis kinetics of hen egg-white lysozyme (HEWL). The extent of fibril formation was monitored via Congo red absorbance at 540(ThT) fluorescence as a function of incubation time. HEWL samples were dissolved in 100 mM glycine buffer (pH 2.0) and incubated at 55°C with agitation of 580 rpm during the course of experiment. The extent of fibril formation was also monitored via Congo red absorption spectra shown in the inset (the arrow indicates the increasing incubation time). Data represent the mean ThT fluorescence intensity of at least 5 independent experiments (n≥5). Error bars represent the standard deviation (S.D.) of the fluorescence measurements.

ThT is a histochemical reagent that stains amyloid deposits and has been widely used as a standard extrinsic fluorescent dye to probe for amyloid fibrils [68]. A significant increase in fluorescence emission intensity is observed when ThT is added to samples containing linear array of β-strands. Upon binding to ordered aggregates of proteins, ThT emits fluorescence intensity that can be measured and is commonly used as a principal index for monitoring the kinetics of fibrillogenesis. As shown in Fig. 1, the change in ThT fluorescence at 485 nm for the control sample of HEWL (without inhibitor added) was found to follow the characteristic nucleation-dependent pathway. The duration of the nucleation phase was approximately 1 hr, and the ThT fluorescence intensity increased rapidly until reaching its (final) equilibration plateau at ∼500 A.U. after approximately 4 hrs of incubation.

Using Congo red binding assay, we further determined if the aggregated species formed in the HEWL sample were fibril-like. Congo red is another well known histological dye, which is widely used to probe for the presence of amyloid fibrils. Tissues containing amyloid deposits stained by Congo red exhibit apple-green birefringence under the cross polarized light [69], [70]. In addition, binding to amyloid fibrils induces an enhanced absorbance and red-shift in the maximum absorbance of Congo red from 490 nm to 540 nm in the absorption spectrum [51], [53]. As demonstrated in the time evolved Congo red absorption spectra of HEWL depicted in the inset of Fig. 1, a considerable increase in absorbance is detected at around 540 nm accompanied by an obvious red-shift of the maximum from 494 to 515 nm, indicating the presence of ordered, β-sheet-rich fibrillar structure in the HEWL sample. Also, the Congo red absorbance difference at 540 nm was found to increase with incubation time, revealing the expansion of fibril growth. Our ThT fluorescence and Congo red absorbance results showed that the aggregates of HEWL formed at acidic pH and high temperature under vigorous agitation exhibited fibril-like characteristics.

**Figure 2:**
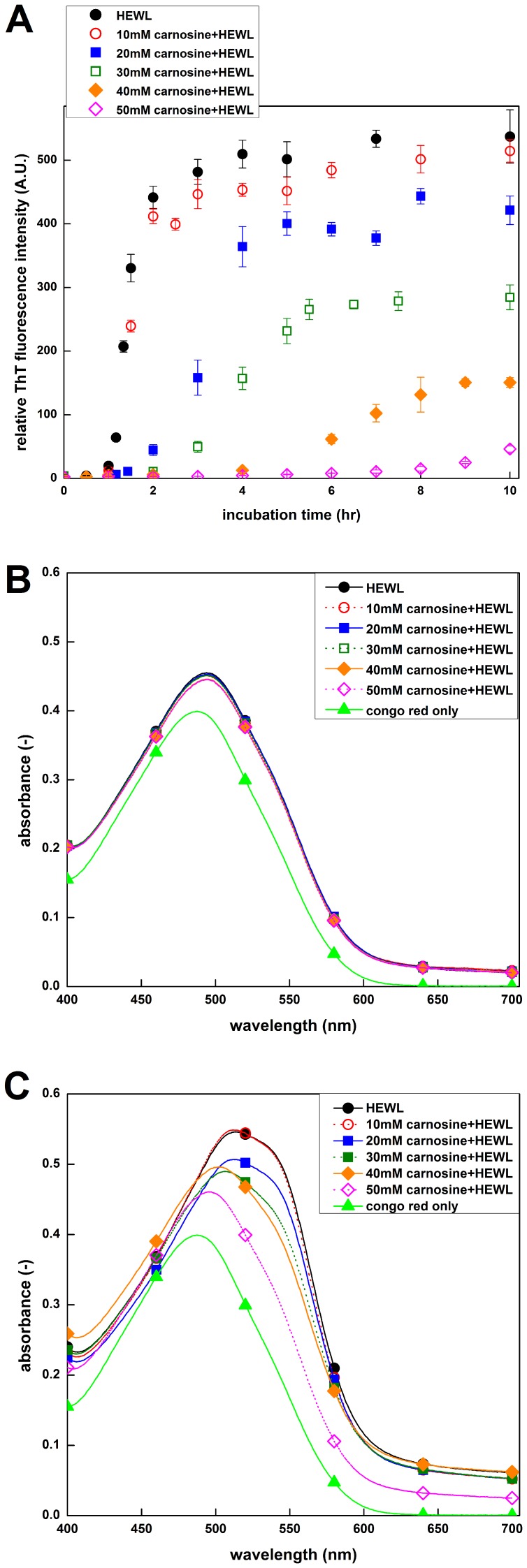
Effects of carnosine on the kinetics of amyloid fibrillogenesis of HEWL. Inhibition of HEWL fibril formation by carnosine in a concentration-dependent manner as revealed by (A) ThT fluorescence and (B and C) Congo red absorbance. The Congo red binding absorbance spectra of HEWL samples were taken at (B) 0 hr and (C) 10 hr of incubation. The samples with and without carnosine were dissolved in 100 mM glycine buffer (pH 2.0) and incubated at 55°C agitated at 580 rpm during the course of the experiment. Each data point represents the mean value of at least 5 independent experiments (n≥5). Error bars denote the standard deviation (S.D.) of the measurements.

To explore the effects of carnosine on HEWL fibrillogenesis, we compared the ThT fluorescence intensities and Congo red absorption spectra of HEWL samples in the presence and absence of carnosine. We demonstrate in Fig. 2A that the change of ThT fluorescence intensity as a function of time in each HEWL sample (with or without carnosine) can be described by a sigmoidal curve, which is composed of a lag phase (without noticeable fluorescence variation), followed by a growth phase (with dramatic fluorescence increase) and a final equilibrium phase where ThT fluorescence reaches a plateau. The final equilibrium ThT fluorescence emission was observed to be negatively correlated with the concentration of carnosine, with the lowest level of ThT fluorescence intensity occurring with 50 mM carnosine. For example, the percentage of reductions calculated by ThT-induced fluorescence at 10 hr of incubation were found to be ∼4.2%, ∼21.6%, ∼47.0%, ∼71.9,% and 91.4% in HEWL samples with 10, 20, 30, 40, and 50 mM carnosine, respectively (the percentage of reduction in ThT fluorescence intensity  = 100%×(ThT fluorescence intensity of HEWL control sample − ThT fluorescence intensity of carnosine-containing HEWL sample)/ThT fluorescence intensity of HEWL control sample).

The Congo red binding assay serves as a complementary measure to detect the presence of amyloid fibril structure in carnosine-containing HEWL samples. Congo red absorption spectra of HEWL with various concentrations of carnosine were found to be similar to that of the HEWL control at the beginning of incubation, as shown in the Fig. 2B. As depicted in Fig. 2C, a marked red shift of the spectral maximum from ∼494 nm to 513 nm, accompanied by an obvious increase in absorbance with an apparent shoulder peak at ∼540 nm, was observed in the absorption spectrum for HEWL with 10 mM carnosine upon 10 hr-incubation. This is akin to the curve obtained for the control sample of HEWL. However, as the concentration of carnosine was raised to 50 mM, apart from the disappearance of shoulder peak at around 540 nm, a significant reduction in both maximum absorbance and red shift of the maximum were also observed, signifying a decrease in the formation of amyloid fibrillar species associated with cross β-pleated sheet. Notably, samples of carnosine alone (at the concentrations used in this study) did not quench ThT fluorescence and alter Congo red binding absorbance (data not shown). Therefore, our ThT fluorescence emission and Congo red binding results, evidently, indicated that carnosine exerts a concentration-dependent attenuating effect on the aggregation/fibril formation of HEWL.

### Electron microscopy analysis of HEWL samples with and without carnosine

**Figure 3:**
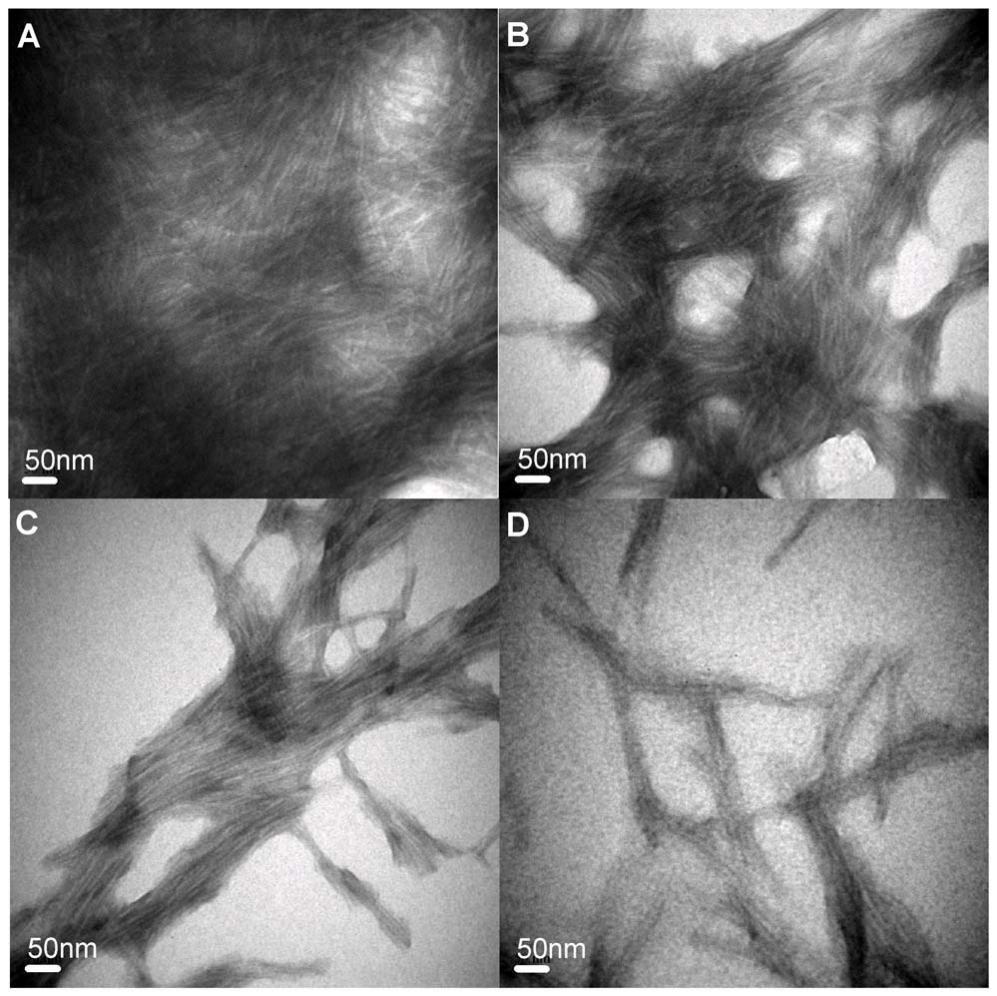
Transmission electron micrographs of HEWL samples with various concentrations of carnosine. Negatively stained electron micrographs of (A) HEWL alone, (B) HEWL co-incubated with 20 mM carnosine, (C) HEWL co-incubated with 40 mM carnosine, and (D) HEWL co-incubated with 50 mM carnosine. HEWL samples in the absence and presence of carnosine were prepared at pH 2.0 and incubated for 10 hr. The scale bar represents 50 nm.

To further investigate the efficacy of carnosine on HEWL fibril inhibition, TEM was used to monitor the morphological features of HEWL samples in the absence and presence of various concentrations of carnosine after 10 hrs of incubation. As displayed in Fig. 3 (top left), the negatively stained micrograph of HEWL control sample contains large quantities of long, un-branched, fibrils of ∼10 nm in diameter and several µm in length, which are characteristics of typical amyloid fibrils. We show in Fig. 3 (top right) that, as compared to the control sample, HEWL sample with 20 mM carnosine exhibits similar structural morphology, but with less fibril formation. HEWL samples co-incubated with higher concentrations of carnosine (40 mM and 50 mM as seen in Fig. 3 bottom left and right, respectively) procured even less fibrils. At the maximum carnosine concentration tested (50 mM), the fibrils that have formed are short and sparsely populated. These TEM observations indicate that carnosine attenuates the amount of HEWL fibrils in a dose-dependent manner. All in all, our ThT fluorescence, Congo red binding, and TEM results suggest that exposure of HEWL to carnosine leads to *bona fide* inhibition/suppression of amyloid fibrillogenesis.

### Conformational transition of HEWL samples with and without carnosine

**Figure 4:**
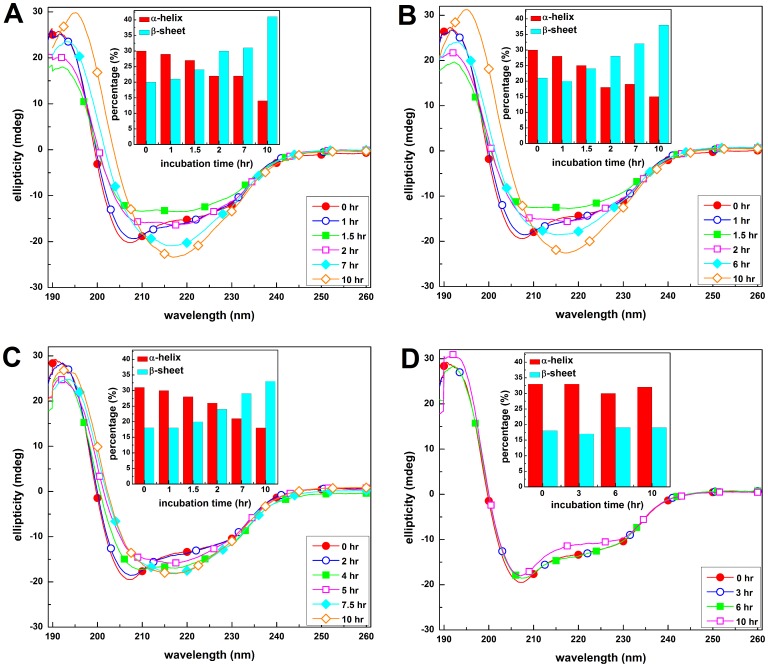
Representative far-UV CD spectra of HEWL samples. The far-UV CD spectra of (A) HEWL alone, (B) HEWL co-incubated with 10 mM carnosine, (C) HEWL co-incubated with 30 mM carnosine, and (D) HEWL co-incubated with 50 mM carnosine. The inset illustrates changes in HEWL secondary structure, showing the contents of α-helix and β-sheet during the fibril formation process.

To examine the effect of carnosine on the conformational (secondary structural) changes of HEWL, we monitored the far-UV CD spectra of HEWL in the absence and presence of carnosine at concentrations of 10–50 mM. As Fig. 4A attests, prior to incubation, a characteristic shoulder at ∼222 nm and a prominent minimum at ∼208 nm are observed in the far-UV CD spectra of all HEWL samples, indicating that the native HEWL is in possession of a predominantly α-helix conformation [21]. However, upon prolonged incubation, the far-UV CD spectrum of the control HEWL sample reveals a marked structural transition, where a prominent change of the secondary structural composition toward a highly amyloidogenic, yet soluble, β-sheet rich conformation, is presented in the CD spectrum as a negative band at 218 nm and a positive ellipticity at around 196 nm. Comparison of the CD spectra, after 10 hrs incubation for HEWL samples treated with 10, 30, and 50 mM carnosine (Figs. 4B, 4C, and 4D, respectively), shows that with increasing concentration of the inhibitor, less pronounced ellipticity values of the negative band at ∼218 nm can be observed. This demonstrates that the structural transition perceived in the HEWL control sample was mitigated upon the addition of carnosine. To quantitatively compare the differences in the conformations among the HEWL samples, the secondary structure contents were estimated by deconvolution of the acquired CD spectra for all HEWL samples using the CONTINLL algorithm available from the DICROWEB server [54]. The resultant time-evolved secondary structure compositions shown in the insets of Figs. 4A–4D indicate that: (1) As expected, the control HEWL sample (Fig. 4A) displayed a time-dependent elevation in β-sheet content that was accompanied by a decrease in α-helix content, suggesting a transition from α-to-β structure (2) The β-sheet content reached the highest level of approximately 41% at 10 hrs after the onset of incubation, which is about two-fold higher than that at time 0 hr. (3) At 10 hr-incubation, the HEWL samples with 30 and 50 mM carnosine (Figs. 4C and 4D) were found to contain a significantly lower β-sheet fraction relative to the control (∼33%, ∼19%, and ∼41% for 30 mM carnosine-HEWL mixture, 50 mM carnosine-HEWL mixture, and the control), demonstrating a positive correlation between the attenuation of β-sheet content and the concentration of carnosine. Overall, our far-UV CD results allow us to conclude that the addition of carnosine is effective in preventing HEWL from undergoing α-to-β transition, which is closely associated with the amyloid fibril-forming propensity.

### The effects of carnosine on the HEWL tertiary structure/surface hydrophobicity

**Figure 5:**
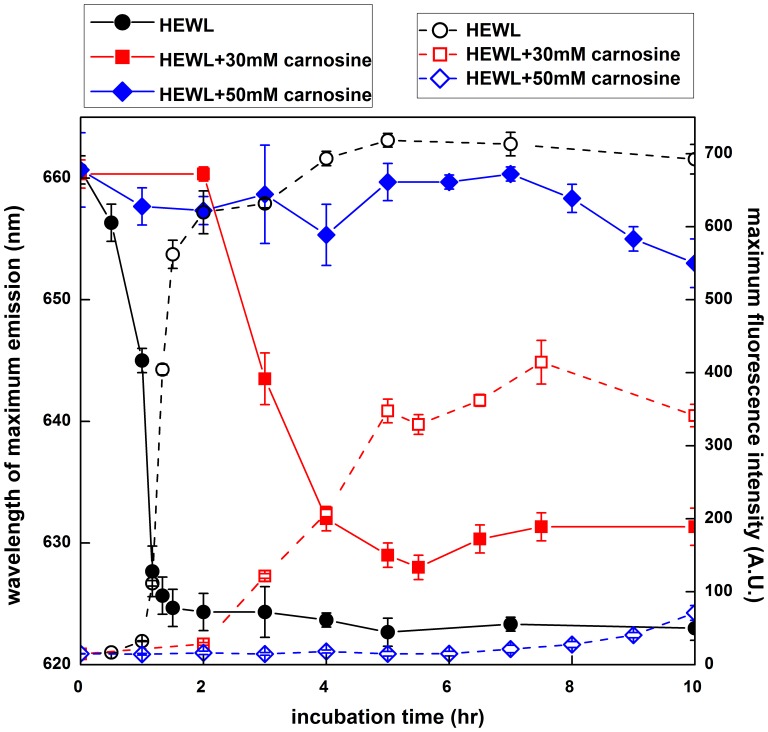
The effects of carnosine on the hydrophobicity of HEWL during incubation. The time-course of the protein surface hydrophobicity was measured by Nile red-binding fluorescence at different incubation times. Data were presented as maximum Nile red fluorescence intensity and wavelength of maximum fluorescence emission taken at various incubation time. Each point represents the average of at least 5 independent measurements (n≥5). HEWL samples were dissolved in 100 mM glycine buffer (pH 2.0) and incubated at 55°C accompanied by agitation of 580 rpm during the course of the experiment (solid symbols represent the wavelength of maximum fluorescence and open symbols are indicative of maximum Nile red fluorescence intensity).

Using the change in surface hydrophobicity as a gauge, the effect of carnosine on HEWL tertiary structure was monitored by the time evolution of Nile red fluorescence emission during the incubation process. Nile red is a nonionic lipophilic fluorescent dye, which has been widely used as a probe to study the intracellular lipid content, polarity of organic solvents, environmental change of biomolecules, and nonionic surfactant microemulsions due to solvatochromism [71]. As illustrated in Fig. 5, the maximum Nile red fluorescence of the control HEWL sample shows no noticeable increase (∼15–30 A.U.) in the first 1 hr of incubation, followed by a dramatic increase from 1 to 2 hr of incubation, and finally reaching an equilibrium plateau (∼700 A.U.) after 4 hr of incubation. In addition, a blue-shift in the wavelength of maximum fluorescence emission (λ_max_), which is indicative of the exposure of hydrophobic clusters, was observed before a significant increase in Nile red fluorescence was detected. The wavelength of maximum fluorescence emission (λ_max_) reached a plateau (from ∼661 nm to ∼623 nm) at 2 hr of incubation, suggesting that the shift of λ_max_ is more sensitive than the enhancement of the Nile red fluorescence emission in probing tertiary structure changes. A similar trend was also observed in the HEWL sample containing 10 mM carnosine (data not shown). However, when a higher concentration of carnosine was added (e.g., 30 or 50 mM), the maximum Nile red fluorescence intensity was found to drop considerably and the blue-shift of λ_max_ was reduced in comparison to that of the control. For instance, upon incubation for 10 hr, the Nile red fluorescence intensity and blue-shift in λ_max_ for carnosine concentration of 30 mM were observed to be ∼341 A.U. and ∼28 nm, respectively, while for 50 mM carnosine were ∼70 A.U. and ∼5 nm, respectively.

### The effect of carnosine on the thermal denaturation behavior of HEWL

The magnitudes of the Gibbs free energy changes (ΔG_U_) of thermal denaturation/unfolding were determined through nonlinear regression against the stability curves obtained. Table 1 presents the calculated values of thermodynamic parameters (ΔG_U_ and T_m_) for all HEWL samples (with and without carnosine). We first compared the thermostability of HEWL in the acidic (pH 2.0) and neutral (pH 7.0) conditions by monitoring the changes average fluorescence emission wavelength (the excitation wavelength  = 280 nm). The thermal denaturation curve of HEWL was found to shift to the left upon changing incubation condition from pH 7.0 to pH 2.0 (shown in Fig. S2 of the supporting information), indicating that incubation at lower pH renders HEWL less stable. The values of T_m_ for HEWL in pH 2.0 and pH 7.0 were determined to be 50.8±4.3°C and 70.9±3.6°C, respectively. For HEWL alone, the ΔG_U_ value at pH 2.0 (−1.11 kcal/mol) was found to be less than that observed at pH 7.0 (+4.92 kcal/mol), implying that a significant smaller thermodynamic barrier to thermal unfolding exists as a result of the pH decrease from pH 7.0 to 2.0.

**Table 1:** Thermodynamic parameters for thermal equilibrium unfolding of HEWL in the absence and presence of carnosine.

Thermodynamic parameter	HEWL (pH 7.0)	HEWL (pH 2.0)	HEWL + 10 mM carnosine (pH 2.0)	HEWL + 30 mM carnosine (pH 2.0)	HEWL + 50 mM carnosine (pH 2.0)
T_m_ (°C)	70.91	50.80	54.01	58.36	66.80
ΔG_U_ (kcal/mol)	4.92	−1.11	−0.24	0.75	3.44

We next examined the thermal denaturation profiles of HEWL in the presence of various concentrations of carnosine. We observed that, relative to HEWL at pH 2.0, carnosine increased the thermal stability of HEWL, and the denaturation curve of HEWL sample with carnosine shifted towards the higher temperature region (shown in Fig. S2 of the supporting information). The calculated T_m_ for HEWL samples with carnosine was higher than that without carnosine. For example, as compared to HEWL alone, the addition of 50 mM carnosine raised the value of T_m_ by ∼16°C (50.80°C for HEWL alone; 66.80°C for 50 mM carnosine-containing HEWL). Moreover, the value of ΔG_U_ was found to increase from −1.11 kcal/mol in the group with no carnosine added to −0.24 kcal/mol, +0.75 kcal/mol, or +3.44 kcal/mol upon exposure to 10, 30, or 50 mM carnosine, respectively.

### Potential mechanism of carnosine inhibition of HEWL fibrillogenesis as revealed by molecular docking

**Figure 6:**
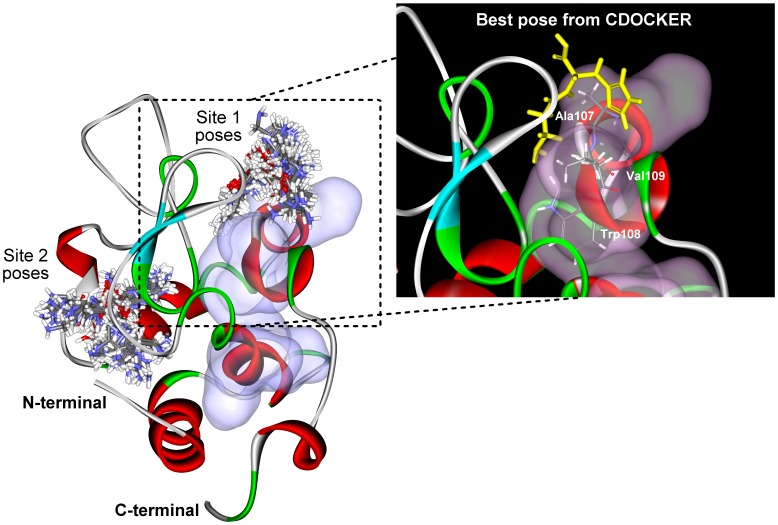
Docking poses of carnosine generated for each of the two predicted binding sites (Sites 1 and 2). The 20-prone regions predicted by the bioinformatics aggregation predictors are surface rendered in violet. Inset: the best docked pose (Pose 1; 0.2 Å yellow-colored sticks) at Site 1 and the three interacting residues (0.12 Å atom-colored sticks) of aggregation-prone region.

To gain molecular insights into how carnosine binds HEWL to prevent aggregation, we first predicted the potential sites responsible for the initiation of amyloid fibril. The results of our consensus aggregation site prediction are depicted in Fig. S3 of the supporting information. A total of two potential aggregation regions, spanning residues N27-C30 and N106-A110, were found based on the primary amino acid sequence of HEWL. These two regions were mapped onto the protein structure and shown in surface representation in Fig. 6. From our initial docking simulations, two potential binding sites (denoted Sites 1 and 2) were found on the protein. However, only the ligand binding poses generated from Site 1 had poses that came within close proximity to one of the potential aggregation regions found by the aggregation site predictors (see Fig. 6). Therefore, only the ligands bound to Site 1 were considered for further analysis.

Table 2 shows the top 10 binding poses of Site 1 based on CDOCKER energy and the protein residues that interacted with carnosine in each of the potential binding modes. Schematic representations of the interactions involved in the binding of the 10 poses are shown in Fig. S4. Based on our prediction, a total of sixteen residues have the potential to be involved in the binding of carnosine to HEWL in a combination of hydrogen bond, polar or charged, and cation-pi interactions. Eleven of these residues (T47, D48, D52, Q57, I58, N59, W62, W63, A107, W108, and V109) interacted with the ligand in all 10 binding poses examined. Interestingly, three of the eleven residues (A107, W108, and V109) were also residues that were predicted to be part of the aggregation-prone region in HEWL (shown in Fig. S3).

**Table 2:** Top 10 binding poses of carnosine.

Pose	CDOCKER energy*	Interaction energy**	Interacting HEWL residues															
No.	(kcal/mol)	(kcal/mol)	N46	T47	D48	D52	Q57	I58	N59	W62	W63	N106	A107	W108	V109	A110	R112	N113
1	−39.9961	−36.7313		√	√	√	√	√	√	√	√		√	√	√		√	√
2	−38.6158	−35.1717		√	√	√	√	√	√	√	√		√	√	√			
3	−38.6133	−35.4767		√	√	√	√	√	√	√	√		√	√	√			
4	−38.5793	−34.3041		√	√	√	√	√	√	√	√	√	√	√	√		√	√
5	−38.4724	−35.1279		√	√	√	√	√	√	√	√		√	√	√			
6	−38.3816	−35.2141		√	√	√	√	√	√	√	√		√	√	√			
7	−38.3501	−34.9482	√	√	√	√	√	√	√	√	√	√	√	√	√	√	√	√
8	−38.2549	−33.4443		√	√	√	√	√	√	√	√	√	√	√	√		√	
9	−38.2086	−35.4429		√	√	√	√	√	√	√	√		√	√	√			
10	−38.1908	−33.6293		√	√	√	√	√	√	√	√	√	√	√	√		√	

*CDOCKER energy  =  Ligand intramolecular energy + Interaction energy

**Ligand-protein interaction energy

### The effect of carnosine on the toxicity of SH-SY5Y cells induced by HEWL amyloid fibrils

**Figure 7:**
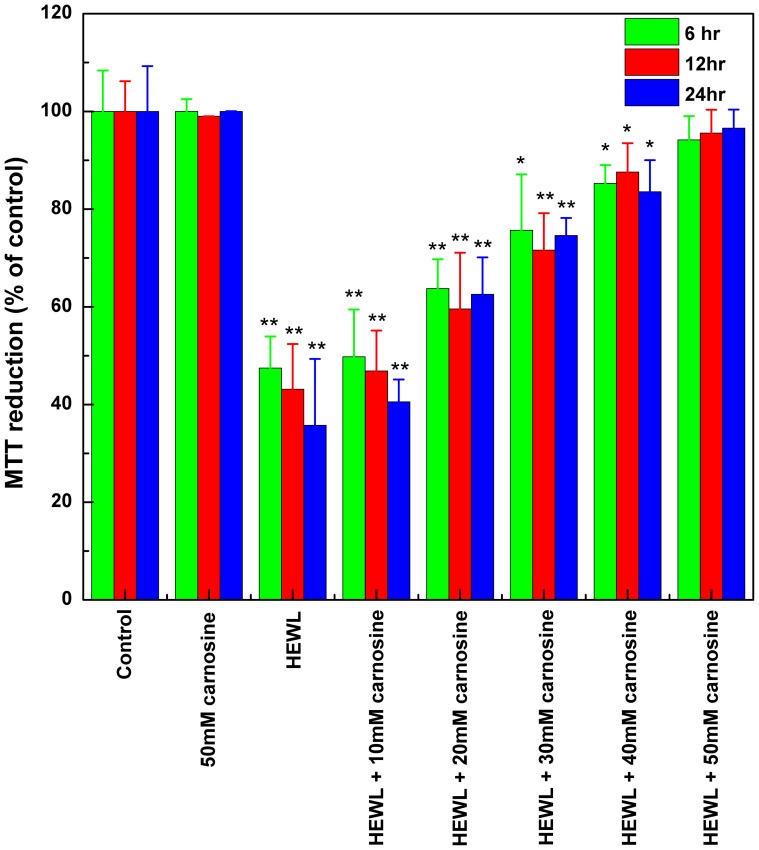
Carnosine's effect on SH-SY5Y cell viability. The cell viability upon exposure to various HEWL samples was measured by MTT reduction. The SH-SY5Y cells were exposed to 50 mM carnosine alone (the negative control) and HEWL samples aged for 10 hr without or with various concentrations of carnosine (10, 20, 30, 40, and 50 mM) for 6, 12, and 24 hr at 37°C in a humidified 5% (v/v) CO_2_/air environment. The data are presented as the percentage of MTT reduced by the cells incubated in media containing 50 mM carnosine alone, HEWL alone, or HEWL with various concentrations of carnosine. The means ± S.D. of at least 10 determinations are shown.

Given its tight association with the metabolic activities/functions of mitochondria in living cells, a decrease in the cell's ability to reduce MTT was taken as an important indicator of cytotoxicity mediated by HEWL fibrils [72]. Presented in Fig. 7 are the effects of carnosine on the HEWL fibril-mediated cytotoxicity in SH-SY5Y cells assessed by MTT reduction assay. As compared with the un-treated cells (control), exposure of SH-SY5Y cells to 10 hr-aged HEWL fibrils lowered the cell viability by ∼53%, ∼57%, or ∼64% for 6, 12, or 24 hr of incubation, respectively. Comparable percentages of MTT reduction (or cell viability) results were obtained in the case of HEWL samples co-incubated with 10 mM carnosine for different periods (∼50% for 6 hr, ∼53% for 12 hr and ∼60% for 24 hr of incubation, as shown in Fig. 7). However, ∼16–17% or ∼28–29% of SH-SY5Y cell viability was restored upon treatment with 20 mM or 30 mM carnosine, respectively. We found that the percentage of MTT reduction or cell viability rose with increasing concentration of added carnosine, with the highest cell viability of ∼94–97% being observed in the 50 mM carnosine-containing HEWL sample (Fig. 7).

### The effect of carnosine on the cell membrane integrity of SH-SY5Y cells treated with HEWL amyloid fibrils as assessed by LDH release/leakage assay

**Figure 8:**
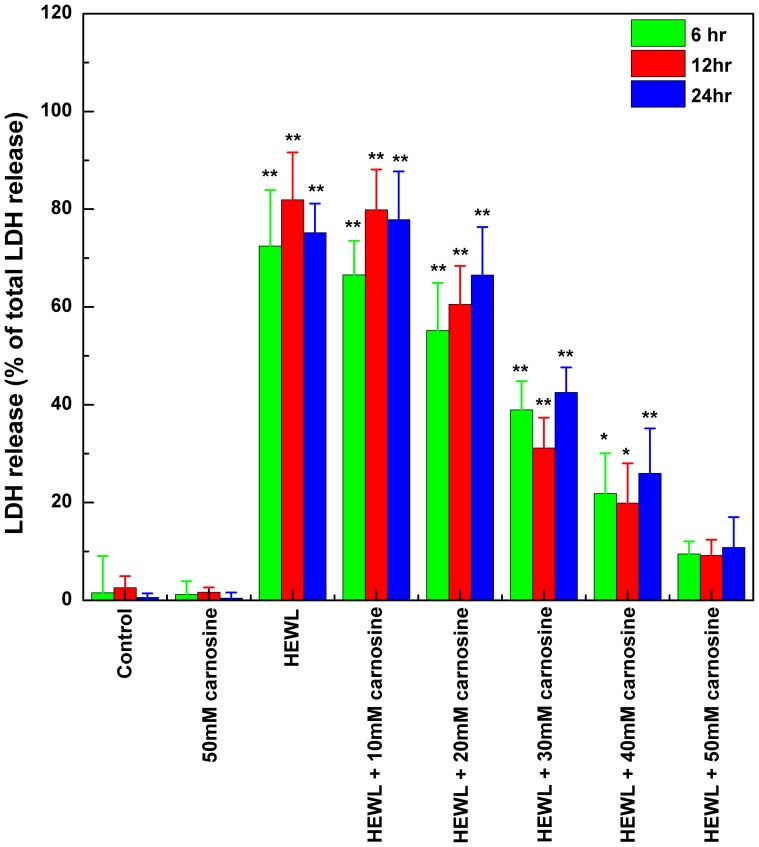
The effects of carnosine on HEWL-induced membrane damage (LDH release into the medium) in SH-SY5Y cells. The cell viability upon exposure to HEWL sample was measured by the LDH release assay. SH-SY5Y cells were incubated with 50 mM carnosine alone (the negative control) and HEWL samples without or with various concentrations of carnosine (10, 20, 30, 40, and 50 mM) for 6, 12, and 24 hr at 37°C in a humidified 5% (v/v) CO_2_/air environment. The percentage of cytotoxicity was evaluated as a ratio of the quantity of LDH released in each sample divided by the total LDH released by the sample of cells treated with lysis buffer. Quantity of released LDH is estimated by the activity of lactate dehydrogenase in the suspension aliquot from the 96-well plates after 30 min incubation with the appropriate substrate solution. Measurements of the means ± S.D. of at least 8 determinations for each sample were obtained at 490 nm.

As a complementary measure of cytotoxicity (or cell viability), the release of the cytosolic enzyme LDH from SH-SY5Y cells after incubation with each HEWL sample (with or without carnosine) for 6, 12, or 24 hr was measured. The amount of LDH release, which is associated with the loss of plasma membrane integrity of living cells, is considered to be closely correlated with the loss of cell viability. As a reference, measurement of the total or maximum LDH (100%) release was obtained from cells treated with lysis buffer solution. We show in Fig. 8 that, when SH-SY5Y cells were treated with 10 mg/mL (∼0.70 mM) of 10 hr-aged HEWL, a significant amount of LDH (approximately 72–82% of LDH release) was detected. Furthermore, the extent of LDH leakage was observed to be negatively correlated with the concentration of carnosine added. While no statistically differences in the levels of LDH release were detected between the groups of HEWL alone and 10 mM carnosine-containing HEWL sample, co-incubation of 10 mg/mL (∼0.70 mM) HEWL with higher concentrations of carnosine (>20 mM) gave rise to a marked reduction in the amount of LDH release. As for the group treated with 50 mM carnosine-HEWL mixture, only a low level of LDH activity was detected in the supernatant demonstrating that majority of the cells were viable with membrane remaining largely intact.

To rule out the possibility that the observed cytotoxic inhibition was mediated by a direct effect of carnosine on the cells, we performed additional MTT reduction and LDH release experiments for the negative control group consisting of SH-SY5Y cells incubated in media containing 50 mM carnosine for 6, 12, or 24 hr. Our results showed that there is almost no difference between the control group and the group treated with 50 mM carnosine (p>0.05), indicating that carnosine has a negligible effect on the cells (as shown in Figs. 7 and 8). Therefore, our observation of the MTT reduction and LDH release results evidently suggest that carnosine possesses a protective effect against HEWL fibril-elicited cytotoxicity on SH-SY5Y cells through its ability to hamper the formation of HEWL amyloid fibrils.

### The effect of carnosine on the pathways of cell death using flow cytometry

**Figure 9:**
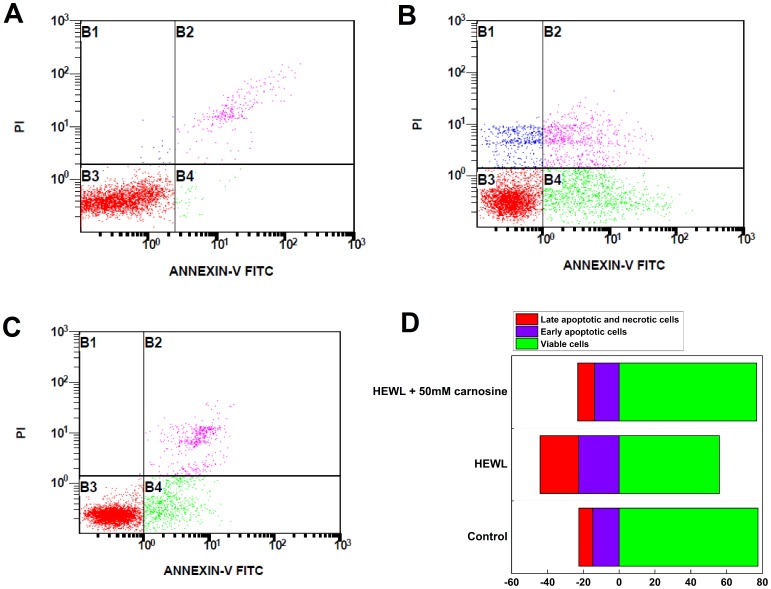
The effects of carnosine on aged HEWL-induced cell deaths analyzed by flow cytometry. Representative intensity dot plots of (A) un-treated SH-SY5Y cells (the un-treated control group); (B) SH-SY5Y cells co-incubated with 10-hr aged HEWL sample; (C) SH-SY5Y cells co-incubated with 10-hr aged HEWL sample containing 50 mM carnosine. The dotted plots are divided into viable (bottom left quadrants; Annexin V-FITC/PI double negative), early apoptotic (bottom right quadrants; Annexin V-FITC positive, PI negative), and late apoptotic/necrotic cells (top right quadrants; Annexin V-FITC/PI double positive) according to Annexin V-FITC and PI fluorescence. (D) Summary of the effects of carnosine on aged HEWL-induced apoptosis. Results are presented as the percentages of viable cells (Annexin V-FITC/PI double negative), early apoptotic cells (Annexin V-FITC positive, PI negative), and late apoptotic/necrotic cells (Annexin V-FITC FITC/PI double positive) according to the PI fluorescence versus Annexin V-FITC fluorescence dotted plots shown in (A), (B), and (C).

To examine how the addition of carnosine affects the mechanism of cell death, we double-stained the SH-SY5Y cells with Annexin-V-FITC and PI. Figs. 9A–9C are the representative dot plot profiles generated by flow cytometric analysis of the cells. As seen in Fig. 9A, while the untreated cells (the control) were primarily Annexin-V-FITC and PI negative, some accidental random staining with both Annexin-V-FITC and PI was consistently noticed, probably attributed to the cell death that occurred as a result of the cell culture operations. Quantitative analysis of the flow cytometry results revealed that treatment of the SH-SY5Y cells with the HEWL sample aged for 10 hr evoked marked staining indicated by the percentage of Annexin V-FITC-positive cells. The proportion of early apoptotic cells (Annexin V-FITC positive/PI negative) increased from ∼14.7% to ∼22.6% accompanied by a 21.5% decrease (from ∼77.5% to ∼56.0%) in viable cell population (Annexin V-FITC negative/PI negative) upon exposure to the 10-hr aged HEWL sample, as shown in Figs. 9B and 9D. The Annexin V-FITC/PI staining results also showed that the addition of the 10-hr aged HEWL sample led to an increase in the percentage of early apoptotic and necrotic cells by ∼7.9% and ∼8.6%, respectively, suggesting that the cell injury triggered by the aged HEWL sample is slightly more necrotic than apoptotic. Comparison of Figs. 9B, 9C, and 9D indicates that the significantly elevated responses in apoptosis and necrosis of SH-SY5Y cells to the 10-hr aged HEWL sample were both depressed upon the addition of 50 mM carnosine. Moreover, as compared with the untreated cells (the control), similar levels/percentages of the four different cell populations (viable cells: negative for Annexin V-FITC and PI signals, early apoptotic cells: Annexin V-FITC signal only, late apoptotic cells: positive for Annexin V-FITC and PI signals, and necrotic cells: positive for Annexin V-FITC and PI signals) were recorded in SH-SY5Y cells treated with the 10-hr aged HEWL sample containing 50 mM carnosine. The flow cytometry results support our above-mentioned MTT reduction and LDH leakage findings that carnosine, in the concentration range used, is capable of reducing cell death triggered by the fibrillar species-containing aged HEWL samples, and that this effect is dosage-dependent.

## Discussion

Multiple hypotheses have been proposed for the etiology and pathogenesis of amyloid diseases, e.g., Alzheimer's disease (AD). Of these, the cholinergic hypothesis states that a loss of cholinergic function associated with acetylcholine (ACh) in the central nervous system contributes substantially to the cognitive decline observed in those with AD [73], [74]. In addition, cholinergic effects have been proposed as a potential causative agent for the formation of plaques and tangles [75]. Another hypothesis, the tau hypothesis focuses on the microtubule binding tau protein in AD as a causative factor in amyloidosis. The abnormal or excessive phosphorylation (hyperphosphorylation) of tau leads to the transformation of normal tau into paired helical filament, (PHF)-tau, which accumulate in neuron as neurofibrillary tangles (NFTs) typically found in histopathological lesions of AD brains [76]. A third hypothesis describes AD as an inflammatory disorder involving microglia, astrocytes, and neurons in the inflammatory processes. Based on this hypothesis, key players that contribute to the inflammatory responses are the complement system, cytokines, chemokines, and acute phase proteins [77], [78]. A fourth hypothesis points to oxidative stress as an etiology of AD. Evidence indicates that AD brains exhibit certain levels of oxidative stress-mediated injury/damage, which is triggered by the chemical reactions between reactive oxygen species and/or free radicals and other molecules (e.g., lipids, proteins, and DNA) [79]. Moreover, there is indirect evidence demonstrating that treatment of antioxidants (e.g., vitamins and polyphenols) delays the progression of AD. These findings suggest that reactive oxygen species and/or free radicals play a role in the development of AD [80]. Lastly, the amyloid cascade hypothesis proposed that the conversion of βamyloid peptide Aβ, which is generated by the proteolytic cleavages of amyloid precursor proteins (APP), into amyloid deposits or toxic assemblies is a causative event in the molecular pathogenesis of the disease. The accumulation/aggregation of Aβ or formation of aggregated Aβ species (e.g., oligomeric and fibrillar Aβ), resulting from an imbalance between generation and clearance, would initiate a cascade of events that ultimately lead to neuronal dysfunction and large scale cell death [81].

While several theories/hypotheses have been proposed, given that numerous lines of evidence from a genetic standpoint indicates that Aβ peptide is the major driving force for protein fribrillogenesis seen in AD, an implication is there that the amyloid pathway is responsible for the pathogenesis of the disease [81], [82]. Thus, the amyloid cascade hypothesis is still deemed the most popular hypothesis contributing to the mechanism of AD and various companies in the biotechnology and pharmaceutical industries have hopped on the bandwagon to direct extensive efforts toward the development of disease-modifying drugs based on the “amyloid cascade hypothesis”.

HEWL is a well-characterized protein and has been proven to possess a propensity for amyloid formation under acidic pH and elevated temperature conditions [22], [28], [83], [84]. HEWL and/or its derivatives are considered as model proteins for the study of amyloid fibrillogenic-relevant behaviors. Previously, investigators have constructed stable and unstable mutants in long helices B and C of HEWL to demonstrate that the long C-helix is associated with the helix to sheet transition during amyloid fibrillogenesis in the acidic environment [85]. Both oligomeric and fibrillar forms of HEWL were shown to exert a dose- and time-dependent cytotoxicity in human neuroblastoma SH-SY5Y cells. While the former was proven to trigger apoptotic cell death, the latter was found to cause necrotic cell death and membrane damage [86]. Others have reported that HEWL fibrils induced extensive aggregation of human erythrocytes and lipid vesicles, suggesting that the HEWL fibril-cellular membrane interaction could be the underlying contributing factor to the disease mechanism [87]. In addition, Dobson's group found that by adding fibrils formed by peptides or full-length HEWL to the native HEWL sample, fibril formation of the native sample was accelerated. The group also proved that the β-domain is significant in full-length HEWL fibril formation [17]. Solubilizing HEWL in highly concentrated ethanol solutions without heating procured amyloid protofilaments rapidly owing to the destruction of the helical and tertiary structures [20]. Results from these and other investigations suggest that partial unfolding is a prerequisite to fibril formation [21], [88]–[94].

A number of researchers have hypothesized that all amyloid-forming polypeptides, regardless of their primary sequence, may display the same structure-specific cytotoxic effects via common mechanism(s) [2], [95], [96]. It has been widely accepted that amyloid proteins are cytotoxic when present in an aggregated form containing mature fibrils, protofibrils, and/or low molecular weight intermediates [97], [98]. Moreover, evidence originating from various studies using cell culture and animal models suggests that the attenuation of amyloid fibrillogenesis appears to be of great benefit to the health of the cells [99], [100]. To that end, strategies that utilize agents/compounds to minimize the formation of aggregated species have been put forth as effective means to alleviate the pathological effects of amyloids or to counteract the progression of amyloid diseases [101]–[103].

Several molecules or compounds, including peptides (or peptide fragments) and non-peptidic small molecules, have been demonstrated to potently attenuate or block the aggregation/fibrillogenesis and/or cytotoxicity elicited by amyloid-forming peptides and/or polypeptides [104], [105]. Typical examples of peptidic molecules that have been reported to exhibit inhibitory activity against amyloid fibril formation are shorter peptide fragments with self-recognition sequences [106]–[108] and small heat shock proteins (e.g., alpha-crystallin Hsp20) [109], [110]. On the other hand, non-peptidic inhibitory molecules consist of a wide range of natural and synthetic aromatic/phenolic ring-containing polyphenols [103], [111]. Molecules such as the sulfonated dye, Congo red, and benzofuran-based compounds have been shown to be effective at reducing fibrillogenesis and cytotoxicity by binding directly to amyloid fibrils [112], [113]. Similar effects were also observed for certain aromatic/phenolic ring-bearing polyphenols (e.g., nordihydroguaiaretic acid and rosemarinic acid), semisynthetic bacteriocidal antibiotics (e.g., rifampicin and its derivatives), surfactants (e.g., di-C6-PC, di-C7-PC, and n-dodecylhexaoxyethylene glycol monoether), and others (e.g., nicotine and trehalose) [96], [114], [115]. Thus, these small molecules could provide a basis for the development of therapeutics for amyloid diseases.

Carnosine (β-alanyl-L-histidine), a naturally-occurring endogenous di-peptide, is present in surprisingly large amounts in long-lived human tissues. Numerous lines of evidence have pointed to the multi-functional significance of carnosine in the human body: (1) serves as a physiological buffering agent [30], [116], [117] and a metal ion (e.g., zinc and copper) chelator [118]–[120]; (2) possessing anti-aging functions [121], [122], and free-radical scavenging activity [123], [124]; (3) capable of delaying senescence [125] and extending the life-span of cultured human fibroblasts [36]; (4) able to kill transformed cells and protect cells against aldehydes and amyloid peptide fragment [126]. Other investigators have concluded that carnosine exhibits a well-documented anti-glycating activity against the glycation of proteins, including low-density lipoproteins, glucose degradation products, esterase, and histones [127]–[130]. Using cardiac aspartate aminotransferase (cAAT) as a model, carnosine was reported to enhance the thermal unfolding and water accessibility of glycated protein species [131]. It also mitigates and/or prevents the alteration in electrophoretic mobility triggered by glyceraldehyde 3-phosphate [132]. Evidence has shown that the methylglyoxal glycation-induced tryptophan fluorescence polarization and scattered light intensity enhancements detected in the aggregated α-crystallin protein were attenuated upon exposure to carnosine [40]. Eye lens opacity in human was also found to be reversed by carnosine [133]. Attanasio and co-workers have made an attempt to explore how both L-form and D-form of carnosine affect the aggregation of bovine α-crystallin [41]. Apart from retaining the chaperone activity of bovine α-crystallin and preventing or reversing the lens opacification, carnosine was observed to have multiple protective roles against bovine α-crystallin fibrillogenesis, including the inhibition of fibril formation and disassembly of preformed fibrils. In addition, using transgenic 3×Tg-AD mice as a model, the amyloid load of mice brain was found to decrease upon dietary supplementation of carnosine [42]. Recently, carnosine has been shown to inhibit the *in vitro* amyloid fibril formation of Aβ(1–42) peptide, probably by disturbing the hydrogen bond network near residues that play crucial roles in fibrillogenesis [43] or by impeding the intermolecular interactions between two key residues (D23 and K28) located at the adjacent Aβ(1–42) monomers [44]. These findings prompted us to further evaluate carnosine's effect on the inhibition of protein fibrillogenesis/aggregation. In this study, we used hen egg-white lysozyme (HEWL), a well-known model protein commonly utilized for the study of protein aggregation, to thoroughly investigate the extent of carnosine fibril/aggregation inhibition on multiple levels.

We began by demonstrating that the formation of HEWL fibrils occurred when the samples were incubated at pH 2.0 and 55°C (see Figs. 1 and 3A). We then tested carnosine for its effects on the *in vitro* amyloid fibrillogenesis of HEWL. ThT fluorescence, Congo red binding, and TEM experiments (see Figs. 2 and 3) all revealed that carnosine exhibits inhibitory activity toward HEWL amyloid fibril formation, and the said inhibitory effect is dependent upon both the incubation period and the carnosine concentration studied (0–50 mM).

The pH-dependent structural stability and/or resistance of proteins to chemical and thermal denaturation have long been explored by other studies to understand how change in pH alters protein conformation [134]–[136]. We also performed equilibrium thermal unfolding experiments to compare the susceptibility of the HEWL structures (in the presence and absence of carnosine) to denaturation/unfolding by heat. A higher structural stability against thermal denaturation upon pH decrease was observed in some proteins [134]. However, in our study, a left-shifted thermal denaturation curve was observed for HEWL at pH 2.0, indicating that a decrease in pH from 7.0 to 2.0 gave rise to a lower resistance to thermal unfolding, which is in agreement with the trend reported in certain proteins [135]. The addition of carnosine also tended to lessen the thermally induced destabilization/unfolding effect of the protein or to augment its thermal stability under the condition of 55°C and pH 2.0 (see Table 1).

Given that a correlation is believed to exist between amyloid fibrillogenesis, secondary structural transitions (from either disordered random structure or α-helix-rich conformation to predominantly ordered β-sheet structures) [137], [138], and surface hydrophobicity, we next recorded the far-UV CD spectra in parallel with Nile red fluorescence spectra of various HEWL samples to monitor the changes in structural conformation due to the addition of carnosine. Our CD results showed that, under the condition of pH 2.0 and 55°C, carnosine mitigated the α-to-β transition observed in the control or aggregating HEWL sample (see Fig. 4), thus preventing HEWL from adopting the conformation comprising primarily β-sheet structure, the suggested secondary structure known to be associated with insolubility and protease resistance [138], [139]. In addition, we observed that the control HEWL and carnosine-containing HEWL samples displayed markedly different Nile red fluorescence spectra. Compared to HEWL alone, exposure to carnosine brought about a decrease in Nile red fluorescence emission and a red-shift in wavelengths of emission maximum (see Fig. 5). This is an implication that the exposure of the hydrophobic regions was appreciably suppressed due to the presence of the dipeptide. It should be noted that, whether the reduction in Nile red fluorescence emission intensity observed in the presence of carnosine is due to the stabilization of HEWL in certain specific conformations with minimized exposure of hydrophobic clusters or merely an indication of the absence of exposed hydrophobic clusters cannot be ascertained from the data obtained so far. However, our results evidently suggest that amyloid fibrillogenesis of HEWL is undoubtedly hampered when the protein is co-incubated with carnosine.

The propensity for a diversity of proteins to form fibrillar aggregates have been a topic intensively explored in both experimental and theoretical studies alike. Through these studies it was found that despite the lack of structural or sequence similarity, these proteins all have the potential to form cross-β structure characteristic of amyloid fibrils [140], and that only certain short sequence stretches on the protein chain is important for the transformation of a protein from its native structure to the ordered fibrillar structure [62], [141], [142]. Although the short residue stretches that are “aggregation-prone” are not similar in sequence, yet they retain inherent property to form β-sheets under suitable conditions [143]. It is said that these aggregation-prone sequence regions are mostly hydrophobic by nature and are usually buried or partially buried within the proteins [59], [142], [144]. The amyloid-promoting regions exhibit intrinsic physicochemical properties that enhance aggregation by stabilizing β-sheet strands and promote further self-assembly of the ordered β-structural motifs into polymers through intermolecular bonding [143]. In the attempt to better understand the physicochemical and structural basis for amyloid formation, various groups have devised *in silico* algorithms to predict potential amyloid nucleation sites based on intrinsic properties of the amino acid sequence itself. As each of these methods make its own assumptions and boasts of its predictive accuracy, we implemented a consensus approach by combining the prediction results of several methods (see **Materials and Methods**) and found two potential aggregation sites, N27-C30 and N106-A110, on HEWL (see Fig. S3).

To understand how carnosine inhibits HEWL amyloid fibril formation, we conducted docking studies and analyzed, theoretically, the binding site of carnosine to HEWL using CDOCKER. Our docking results showed that carnosine initially binds to an aggregation-prone region through interactions with the following hydrophobic residues: A107, W108, and V109. Based on the best binding mode (inset of Fig. 6 and Pose 1 in Fig. S4) derived from the docking simulation, the interaction pattern involving these residues include hydrogen bonding with A107 while interactions with W108 and V109 were charged or polar. Other residues that prevalently form hydrogen bonds with carnosine were D48, Q57, and W63. In addition to the hydrogen bonds and charged or polar interactions, Pose 1 rose above all others owing to an additional cation-pi interaction that existed between the imidazole ring of carnosine and R112 of HEWL, which may serve to stabilize the ligand-protein complex. Based on our molecular docking results, we believe that carnosine inhibits HEWL aggregation by initially blocking aggregation-prone site mainly through interactions with three residues: A107, W108, and V109.

To test that carnosine's effect against HEWL fibrillogenesis is not just a generalized phenomenon of dipeptide molecules, we have performed a couple of control experiments, in which di-alanine, di-histidine, or di-glycine at 50 mM was incubated with HEWL and samples were taken at various time points for ThT binding assay. As illustrated in Fig. S5, the inhibitory potency against lysozyme fibril formation shows the following order: carnosine > di-alanine > di-histidine > di-glycine. The reasoning behind this order of effect can be explained by the relative location of the potential aggregation-prone region on HEWL, the bulkiness of the inhibitor's sidechain, and the extent of interactions involved in binding carnosine to HEWL to block the aggregation-prone site from initiating the process of amyloid fibrillogenesis. Through our bioinformatic prediction and docking studies, we found that the region spanning residues N106 ∼ A110 to be an aggregation-prone region and that residues A107 ∼ V109 (which interact with carnosine) are situated at the opening of the HEWL catalytic cleft. As can be seen from the best docking pose shown in Fig. 6, the binding position of carnosine is at the mouth of the catalytic cleft with the β-alanine inserted into the cleft while the bulky imidazolium group of histidine is left out sitting at the lip of the cleft. This orientation maximizes the number of residues and atomic interactions involved in binding carnosine to HEWL, thus stabilizing the protein-ligand complex. Whereas histidine plays a supporting role in anchoring carnosine to the mouth of the cleft, di-alanine is small enough to enter the cleft and may be less specific in binding to the aggregation-prone region. However, based on our docking results, it seems that alanine is involved in majority of the interactions observed between carnosine and HEWL (see Fig. S4 of the supporting information); therefore, it is likely that once di-alanine found its way to the aggregation-prone site, it is still capable of exerting an anti-fibrillogenic effect on HEWL to some extent. Having said that alanine participates in most of the atomic interactions observed between carnosine and HEWL, it is understandable that di-histidine has less of an inhibitory effect than di-alanine in preventing HEWL fibril formation. Based on past studies, it has been found that two cationic groups in protein or peptide residues can form contact pairs despite both bearing positive charges [145], [146]. Since the imidazole group in histidine (pK∼6) is protonated in low pH condition, the dipeptide assumes a double positive charge under the experimental setting explored in our study. According to Heyda *et. al.* (2010) [147], the cationic groups in di-histidine and other histidine-based dipeptides exhibit attractive interactions regardless of the unfavorable Coulomb repulsion force. Due to the covalent linkage between the two histidine residues, the contact pairing of the imidazolium side chains preferably assumes a parallel or off-parallel stacking orientation, which restricts the extent of separation between the two groups. This steric constraint placed on di-histidine meant that the dipeptide nearly folds back on itself to form cationic pairing, in essence, reducing the accessibility of atomic interactions that can potentially form with HEWL if it were laid out with the two imidazolium groups spread out side-by-side. As for di-glycine, the dipeptide of the smallest amino acid glycine, our ThT fluorescence results demonstrate that there is almost no difference in the ThT fluorescence signal between the HEWL alone sample (the control) and the di-glycine-containing HEWL sample, suggesting that di-glycine does not exert an inhibitory action toward HEWL fibrillogenesis.

Having established that carnosine has an inhibitory effect on HEWL amyloid fibrillogenesis, we next set out to investigate the effects of carnosine on cell toxicity in the presence of HEWL aggregates. Previously, it was suggested that amyloid peptides/proteins demonstrate neuro-cytotoxicity in their aggregation states [148]. We, therefore, subjected SH-SY5Y human neuroblastoma cells to co-incubation with 10-hr aged HEWL sample that has previously been shown by our group to have distinct features characteristic of amyloid fibril. These features include: adopting the β-sheet-rich conformation (∼41%), having a high affinity for Congo red, emitting high level of ThT fluorescence, and displaying fibrillar morphology based on TEM (see Figs. 1, 2, 3, and 4). Prolonged exposure to the 10-hr aged HEWL sample considerably decreased the viability of the SH-SY5Y cells. Our MTT reduction results in concert with the LDH release findings allow us to conclude that the significant cellular damage elicited by the aforesaid fibrillar form of HEWL may be inhibited by carnosine in a concentration-dependent manner (see Figs. 7 and 8). We also carried out flow cytometric analysis, which revealed that co-incubation with carnosine dipeptide also attenuated the extent of apoptotic/necrotic cell death induced by the HEWL sample containing fibrillar species (see Fig. 9). These data are well corroborated with other findings on the protection of REB4 cells by carnosine against cytotoxicity triggered by fibrillar Aβ(25–35) [149].

Both MTT and Annexin V/PI apoptosis assay detect cell viability, but on a different level. Based on the results of our MTT assay, it can be seen that the pH 2-fibrillized HEWL impaired the metabolic activity or health of the cells by ∼52–64%. However, with the increased dosage of carnosine, we began to see a recovery of metabolic activity in the SH-SY5Y cells until close to ∼95% recovery is achieved in the condition where 50 mM carnosine was added. As MTT assay is only capable of indicating the overall well-being of a cell population rather than pointing to the more specific states of cell death (which can be differentiated by the Annexin V/PI apoptosis assay), we therefore, performed Annexin V/PI apoptosis assay for a more complete picture of cell viability. Consistent with previous reports that necrosis is associated with cytotoxicity elicited by the fibrillar form of HEWL [86], our Annexin V/PI apoptosis assay also shows that the cytotoxicity induced by the pH 2.0-fibrillized HEWL is slightly more necrotic, with an increase of 8.6% necrotic versus 7.9% early apoptotic cells, when comparing the changes in Figs. 9A, 9B, and 9D. It is noteworthy to mention that carnosine protects the cells from both necrosis and apoptosis indiscriminately by maintaining the cell populations at similar levels as the untreated control group (Figs. 9A, 9C, and 9D).

In conclusion, the research presented here examines how carnosine influences *in vitro* protein fibril formation via several spectroscopic techniques, TEM, thermally induced unfolding analysis, and molecular docking. We observed that carnosine has a concentration-dependent inhibitory activity against β-sheet formation and *in vitro* HEWL fibrillogenesis, with the best inhibition level observed at carnosine concentration of 50 mM. Moreover, thermally induced equilibrium denaturation analysis revealed that HEWL exhibited enhanced stability upon the addition of 50 mM carnosine. The dipeptide exerts its fibril-inhibitory effect by initially binding to a partially exposed aggregation-prone region of HEWL, thereby protecting the protein from amyloid fibril nucleation. In addition, through the MTT reduction and LDH release assays, we were able to show that carnosine attenuated the decline in SH-SY5Y cell viability induced by the aged HEWL samples containing predominantly HEWL fibrils. The protective action of carnosine against HEWL fibril induced neural cell death is through both anti-apoptotic and anti-necrotic activities elicited in the neural-like cells, as evaluated by the flow cytometric analysis. While the amyloid fibrillogenesis of hen lysozyme, especially at acidic pH and elevated temperature, is rarely observed *in vivo* and further investigation is warranted to fully decipher the detailed mechanism by which carnosine may retard the extent of HEWL fibrillogenesis/aggregation and reduce the cytotoxicity derived from HEWL fibrils, we believe the results from the current work are still informative and can provide a model system for future work in this subject matter. It is worth re-emphasizing that carnosine is currently being used as a component in eye drops for the prevention and treatment of cataract in Europe [150]. Along this line, some of the knowledge gained from this work indicates that carnosine may also be a promising strategy for ameliorating the progression of protein aggregation diseases such as amyloidoses.

## Supporting Information

Figure S1 **The time dependence backbone alpha carbon root-mean-square deviation (RMSD; nm) of HEWL.** The 20 ns simulation was performed in pH 2.0 and 55°C.
(TIF)

Figure S2 **Thermal denaturation curves of HEWL samples.** The thermal denaturation curves are obtained by plotting fractions of denatured/unfolded HEWL under different conditions (solid black circles: HEWL at pH 7.0; solid red circles: HEWL at pH 2.0; solid blue squares: HEWL+10 mM carnosine at pH 2.0; solid green squares: HEWL+30 mM carnosine at pH 2.0; solid orange diamonds: HEWL+50 mM carnosine at pH 2.0) against the temperature.
(TIF)

Figure S3 **Consensus aggregation site prediction revealing two potential aggregation regions on the primary sequence of HEWL.**
(TIF)

Figure S4 **Two-dimensional schematic representation of the top 10 potential binding modes for carnosine (Poses 1–10).**
(TIF)

Figure S5 Time-evolved ThT fluorescence emission of lysozyme samples containing 50 mM of di-glycine, di-histidine, di-alanine, and carnosine.
(TIF)
