# Supplementary figures and images for: Carnosine's Effect on Amyloid Fibril Formation and Induced Cytotoxicity of Lysozyme
Source: PLoS One. 2013 Dec 11;8(12):e81982. doi: 10.1371/journal.pone.0081982 (PMC3859581; doi:10.1371/journal.pone.0081982)

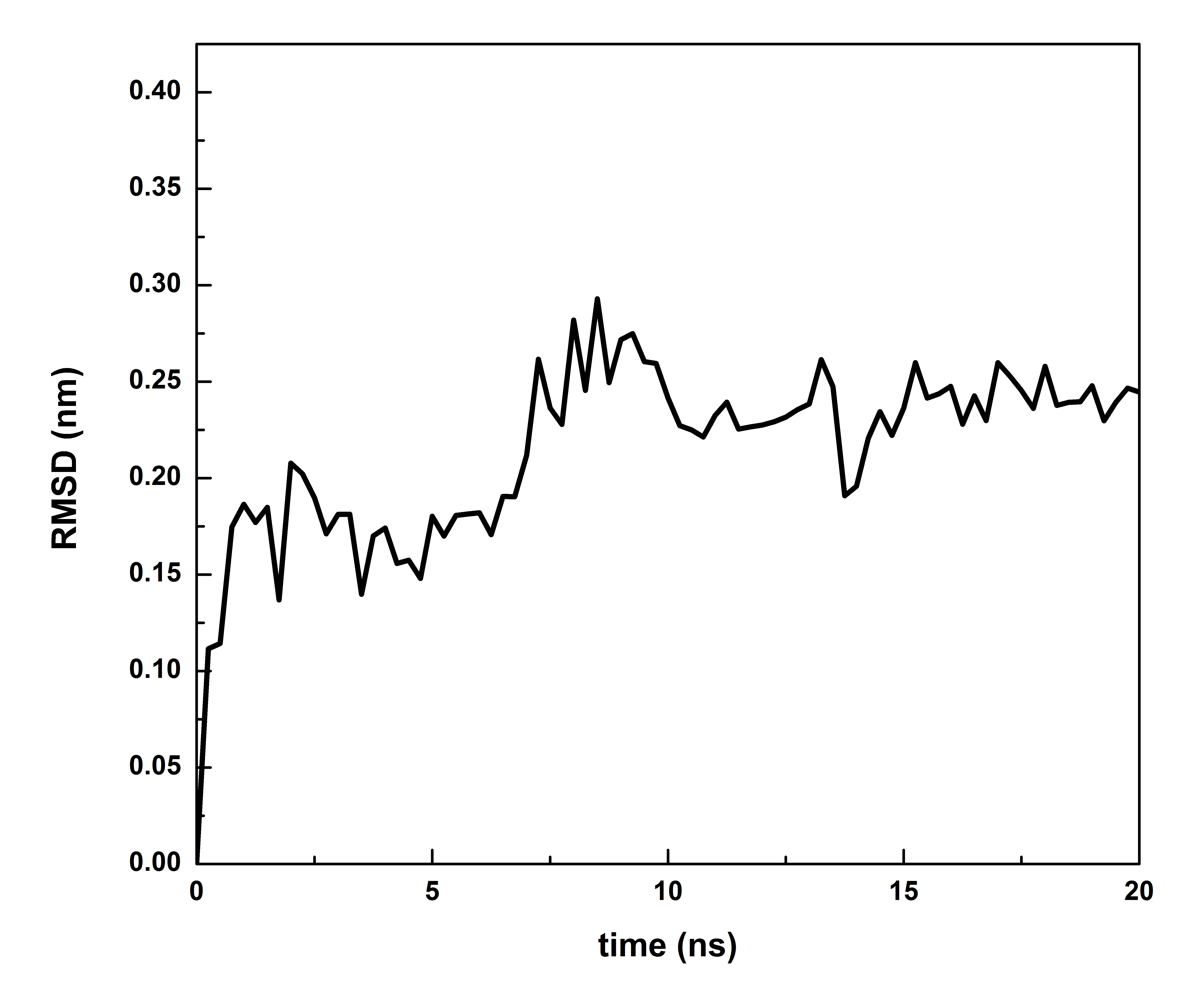

Supplement: Figure S1 — The time dependence backbone alpha carbon root-mean-square deviation (RMSD; nm) of HEWL. The 20 ns simulation was performed in pH 2.0 and 55°C. (TIF) [file pone.0081982.s001.tif]

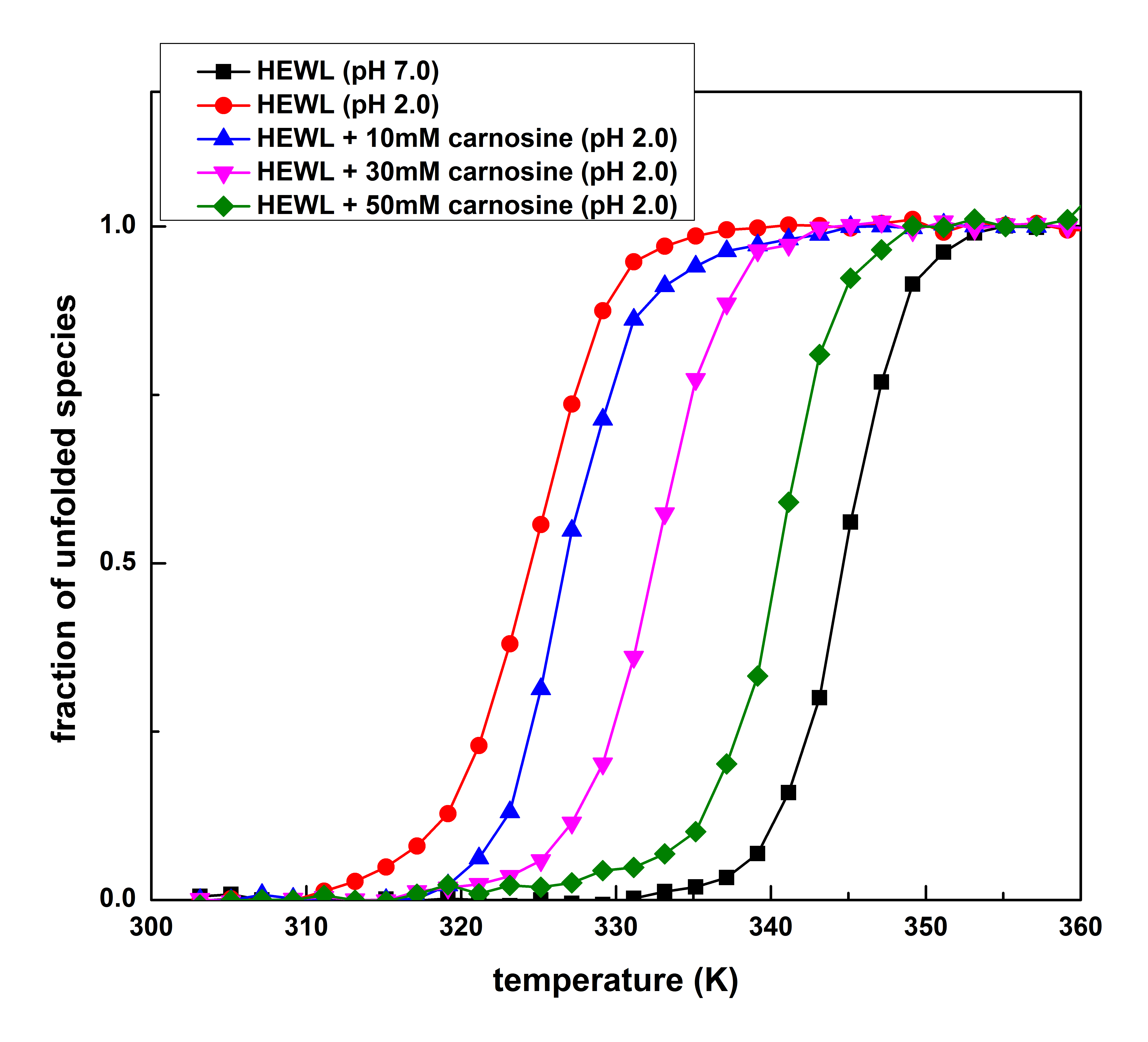

Supplement: Figure S2 — Thermal denaturation curves of HEWL samples. The thermal denaturation curves are obtained by plotting fractions of denatured/unfolded HEWL under different conditions (solid black circles: HEWL at pH 7.0; solid red circles: HEWL at pH 2.0; solid blue squares: HEWL+10 mM carnosine at pH 2.0; solid green squares: HEWL+30 mM carnosine at pH 2.0; solid orange diamonds: HEWL+50 mM carnosine at pH 2.0) against the temperature. (TIF) [file pone.0081982.s002.tif]

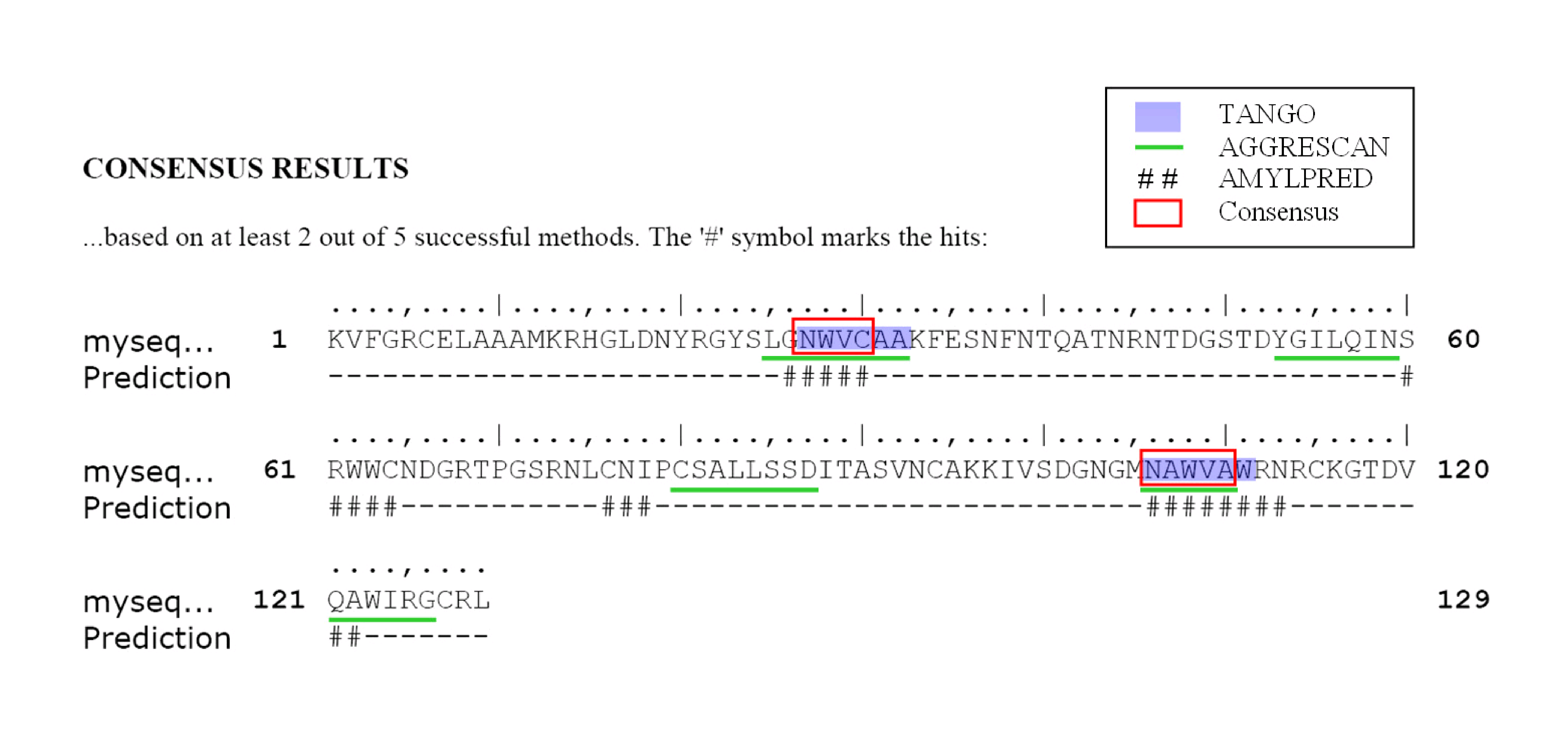

Supplement: Figure S3 — Consensus aggregation site prediction revealing two potential aggregation regions on the primary sequence of HEWL. (TIF) [file pone.0081982.s003.tif]

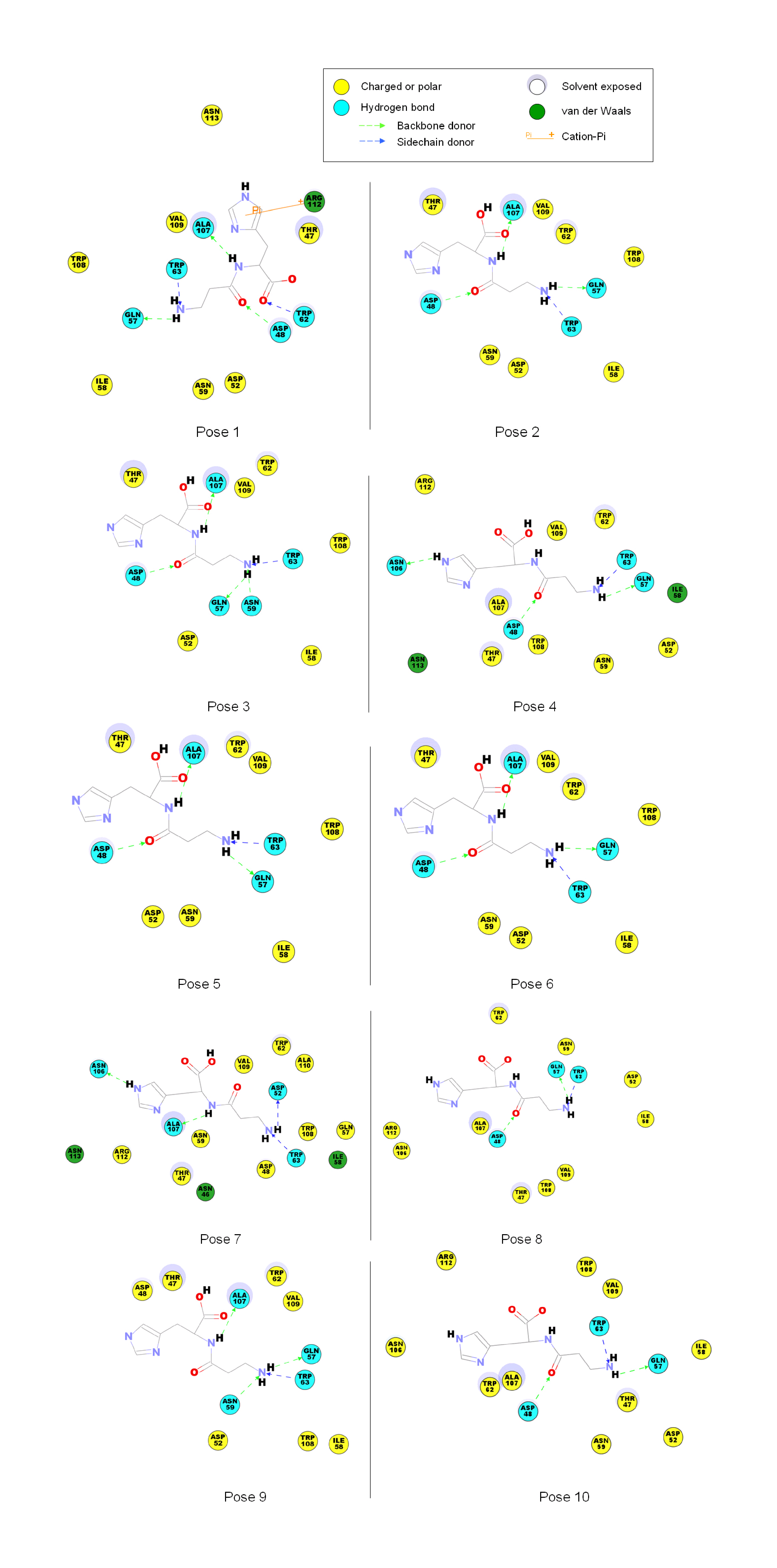

Supplement: Figure S4 — Two-dimensional schematic representation of the top 10 potential binding modes for carnosine (Poses 1–10). (TIF) [file pone.0081982.s004.tif]

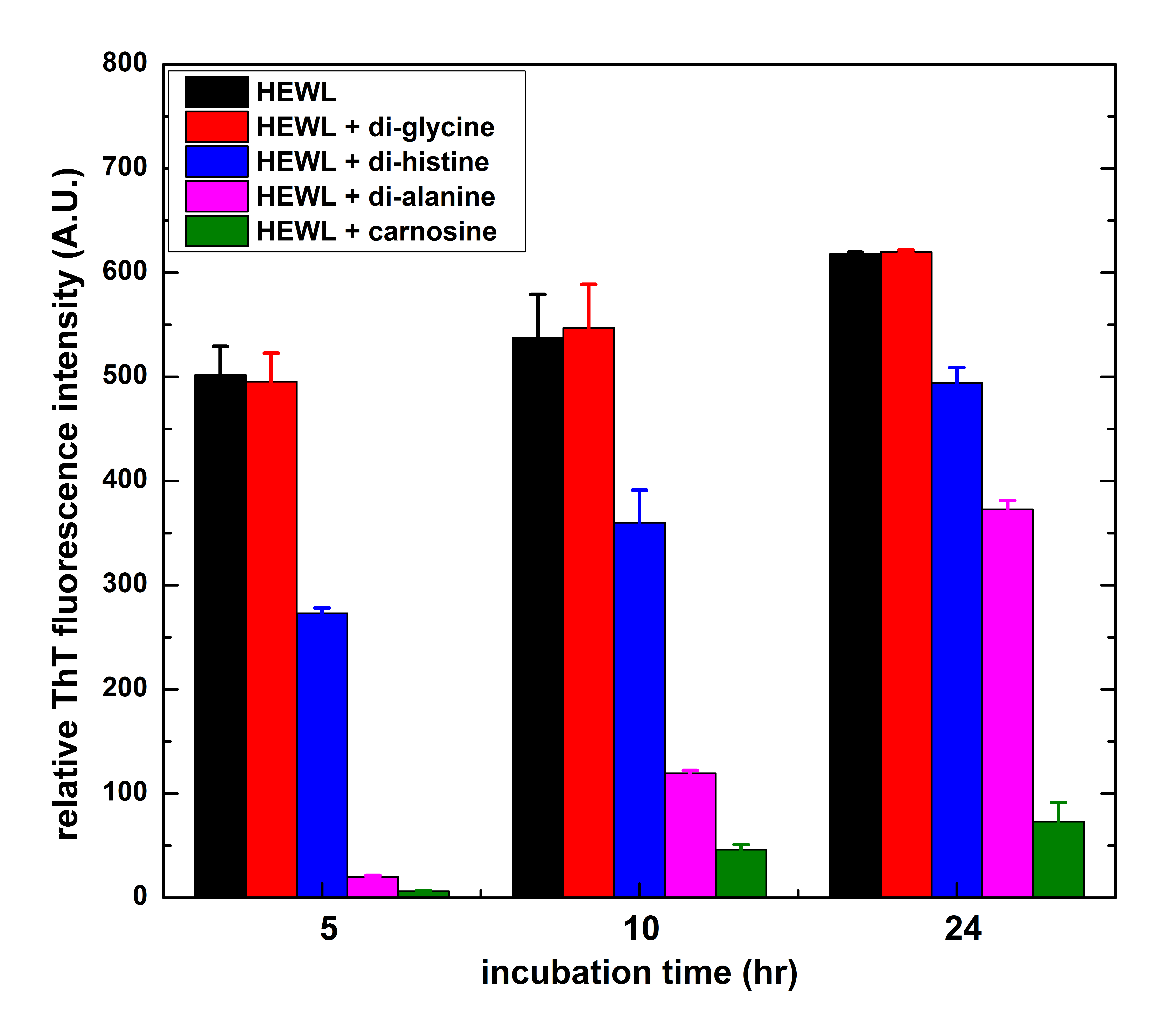

Supplement: Figure S5 — Time-evolved ThT fluorescence emission of lysozyme samples containing 50 mM of di-glycine, di-histidine, di-alanine, and carnosine. (TIF) [file pone.0081982.s005.tif]
